# Expansion microscopy reveals characteristic ultrastructural features of pathogenic budding yeast species

**DOI:** 10.1242/jcs.262046

**Published:** 2024-09-09

**Authors:** Md Hashim Reza, Srijana Dutta, Rohit Goyal, Hiral Shah, Gautam Dey, Kaustuv Sanyal

**Affiliations:** ^1^ Molecular Mycology Laboratory, Molecular Biology and Genetics Unit, Jawaharlal Nehru Centre for Advanced Scientific Research, Jakkur, Bengaluru 560064, India; ^2^ Cell Biology and Biophysics, European Molecular Biology Laboratory, Heidelberg 69117, Germany; ^3^ Department of Biological Sciences, Bose Institute, Unified Academic Campus, EN-80, Sector V, Bidhan Nagar, Kolkata 700091, India

**Keywords:** Ultrastructure expansion microscopy, *Candida albicans*, Spindle pole bodies, Nucleolus, Microtubules, Fungal pathogens

## Abstract

*Candida albicans* is the most prevalent fungal pathogen associated with candidemia. Similar to other fungi, the complex life cycle of *C. albicans* has been challenging to study with high-resolution microscopy due to its small size. Here, we employed ultrastructure expansion microscopy (U-ExM) to directly visualise subcellular structures at high resolution in the yeast and during its transition to hyphal growth. N-hydroxysuccinimide (NHS)-ester pan-labelling in combination with immunofluorescence via snapshots of various mitotic stages provided a comprehensive map of nucleolar and mitochondrial segregation dynamics and enabled the resolution of the inner and outer plaque of spindle pole bodies (SPBs). Analyses of microtubules (MTs) and SPBs suggest that *C. albicans* displays a side-by-side SPB arrangement with a short mitotic spindle and longer astral MTs (aMTs) at the pre-anaphase stage. Modifications to the established U-ExM protocol enabled the expansion of six other human fungal pathogens, revealing that the side-by-side SPB configuration is a plausibly conserved feature shared by many fungal species. We highlight the power of U-ExM to investigate subcellular organisation at high resolution and low cost in poorly studied and medically relevant microbial pathogens.

## Teaser

**Summary:** Expansion microscopy enables the visualisation of cell cycle-dependent distinct organellar organisation in the human pathogen *Candida albicans*, and is generalised to the study of other pathogenic yeasts.

## INTRODUCTION

Since the first documented use of lenses to discover microbial life forms by Hooke and Leeuwenhoek during 1665–1683 (Gest, 2004), various advancements have been brought about to improve the resolution of imaging. Conventional fluorescence microscopy is limited by low spatial resolution due to the diffraction limit of light that ranges from 200–300 nm laterally. Additionally, imaging cellular subcompartments of fungi is limited due to the smaller-sized organelles, often beyond the diffraction limit of conventional fluorescence microscopes. The advent of super-resolution microscopy techniques, like structured illumination microscopy (SIM), photo-activated localisation microscopy (PALM) and stochastic optical reconstruction microscopy (STORM) have been able to achieve a resolution in the range of 50–120 nm (Betzig et al., 2006; Rust et al., 2006). The complexities associated with image acquisition and processing, coupled with the high cost of the microscopes, limit the throughput and benefits of super-resolution microscopy. The discovery of expansion microscopy (ExM), which relies on the isotropic physical expansion of biological samples rather than altered optics, enables super-resolution imaging using a diffraction-limited microscope (Chen et al., 2015). To date, the application of ExM to visualize ultrastructure in fungi is limited only to a few species including *Saccharomyces cerevisiae*, *Schizosaccharomyces pombe*, *Aspergillus fumigatus* and *Ustilago maydis* (Chen et al., 2021; Götz et al., 2020; Hinterndorfer et al., 2022). This is largely due to a complex cell wall composition, which prevents uniform expansion of the cell content in fungal species.

A common human microbiome resident, *Candida albicans* can transition from its otherwise commensal lifestyle to a pathogenic state (Mayer et al., 2013). *C. albicans* can switch between various morphotypes including yeast and hyphae. The yeast–hyphal transition is necessary for *C. albicans* pathogenicity, enabling tissue invasion and subsequent tissue damage during candidiasis (Sudbery et al., 2004; Lohse and Johnson, 2009). Additionally, plasticity with respect to ploidy, single nucleotide polymorphism (SNP), loss of heterozygosity (LOH), copy number variations (CNVs) and chromosomal instability (CIN) events all make *C. albicans* a successful pathogen (Legrand et al., 2019; Selmecki et al., 2010). Of late, *C. albicans* has gained significant attention as a model organism for the study of nuclear division owing to attributes such as a dynamic genome, cryptic heterochromatin machinery (Sreekumar et al., 2019) and unique centromere properties (Legrand et al., 2019; Guin et al., 2020). Although kinetochore proteins and their organisation have been studied in *C. albicans*, information regarding the spatial and molecular organisation of spindle pole bodies (SPBs) is largely lacking. SPBs, the functional equivalent of metazoan microtubule-organising centres (MTOCs), nucleate nuclear and astral microtubules (aMTs), which segregate sister chromatids and position the spindle during the cell cycle, respectively (Markus et al., 2012; Palmer et al., 1992; Shaw et al., 1997; Sullivan and Huffaker, 1992). Positioning and alignment of the mitotic spindle along the polarity axis is vital for asymmetric cell division. The fungal kingdom displays remarkable diversity in the positioning of the mitotic spindle during the pre-anaphase stage of the cell cycle (Finley et al., 2008; Kopecka et al., 2001; Kozubowski et al., 2013; Maekawa et al., 2017; Markus et al., 2012; Martin et al., 2004; Mochizuki et al., 1987; Pereira et al., 2001; Winey and Bloom, 2012; Yamaguchi et al., 2009). In *S. cerevisiae*, the nucleus migrates to the bud neck with the mitotic spindle aligned to the bud axis at the pre-anaphase stage to achieve chromosomal division (Markus et al., 2012; Pereira and Yamashita, 2011; Winey and Bloom, 2012). However, both the nucleus and the mitotic spindle are positioned away from the bud neck in the pre-anaphase cells of *C. albicans* (Finley et al., 2008; Martin et al., 2004). This difference in SPB-dependent regulation of chromatid segregation further hints towards a distinctive feature of *C. albicans* cell biology that requires further exploration.

In this study, we established a working cell expansion protocol for *C. albicans* and succeeded in visualising subcellular structures in both planktonic yeast cells and hyphal germ tubes using N-hydroxysuccinimide (NHS)-ester pan-labelling (M'Saad and Bewersdorf, 2020) combined with immunofluorescence (IF). We provide a characterisation of the mitotic cycle at ultrastructural resolution, revealing a unique configuration of SPBs in *C. albicans*. Finally, we demonstrate the applicability of U-ExM to six other important fungal pathogens.

## RESULTS

### U-ExM reveals changes in the cellular ultrastructure during the yeast–hyphal transition in *C. albicans*

**Fig. 1.**
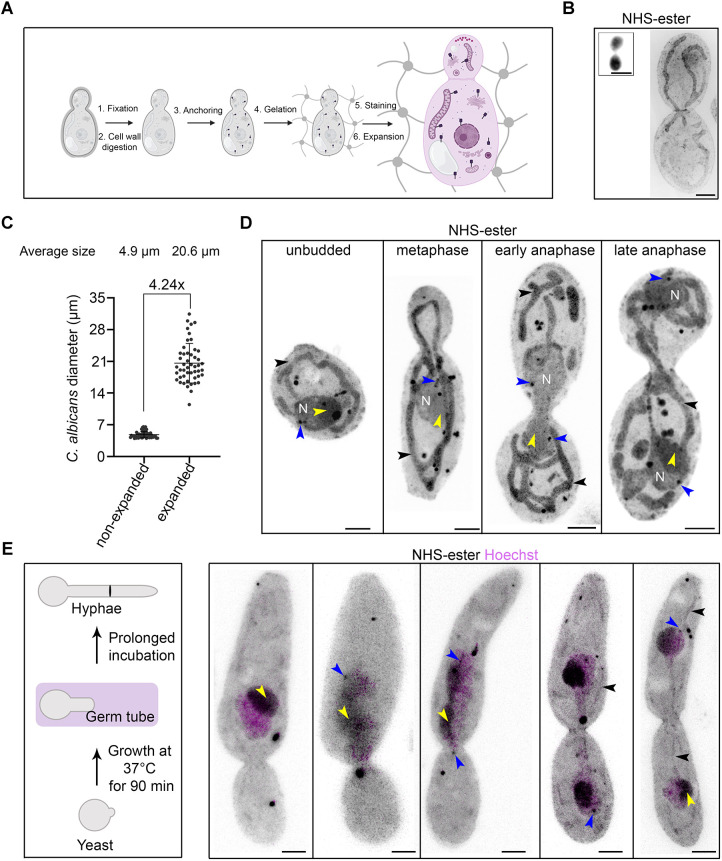
**Pan-labelling of proteome displays subcellular organisation in expanded *C. albicans* cells.** (A) Schematic displaying different steps of ultrastructure expansion microscopy (U-ExM) protocol in *C. albicans*, which includes fixation (1), cell wall digestion (2), anchoring (3), gelation (4), staining (5), and expansion (6). (B) Representative confocal image of *C. albicans* cells post-expansion, pan-labelled with NHS-ester and displayed as maximum intensity projections. The inset shows a non-expanded cell. Scale bars: 5 µm. (C) Scatter plot displaying the 4.24× expansion factor based on measurement of the diameter (longer axis) of unbudded cells of *C. albicans* before (*n*=54) and after (*n*=49) expansion. Error bars show mean±s.d. (D) Representative images of *C. albicans* pan-labelled with NHS-ester showing subcellular organisation at various stages of the cell cycle. The black and blue arrowheads represent mitochondria and SPBs, respectively. The nucleus is marked with N and the nucleolus is marked with a yellow arrowhead. Scale bars: 5 µm. (E) Schematic showing morphogenetic changes during the yeast-to-hyphal transition. Cells at the germ tube stage (magenta) were taken forward for U-ExM. Representative images of *C. albicans* pan-labelled with NHS-ester (grey) and co-stained with Hoechst 33342 (magenta) showing subcellular organisation during cell division after hyphal induction (*n*=2). The black, blue and yellow arrowheads represent mitochondria, SPBs and the nucleolus respectively. Scale bars: 5 µm. All images representative of three experimental repeats. For U-ExM images, scale bars have not been rescaled for the gel expansion factor.

In most organisms with a cell wall, the nanoscale isotropic expansion that is necessary for the U-ExM technique relies heavily on a post-fixation strategy to evenly digest the wall. Therefore, we optimised the digestion of the cell wall in the human fungal pathogen *C. albicans*. Log-phase chemically fixed cells were digested with Zymolyase 20T in a buffer containing 1.2 M sorbitol to prevent cell lysis. Post-digestion, cells were subjected to anchoring, followed by gelation, denaturation and expansion (Fig. 1A). The gels were stained with an NHS-ester compound which non-specifically labels the proteome and enables visualisation of the protein density map of a cell (Kozubowski et al., 2013) (Fig. 1B). Next, we examined the degree of isotropic expansion and calculated the expansion factor. We measured the diameter of the *C. albicans* cell before (4.85 µm) and after expansion (20.6 µm) revealing that *C. albicans* could be expanded ∼4.24-fold (Fig. 1C). This is in line with the reported fold expansion for *S. cerevisiae* and *S. pombe* (Hinterndorfer et al., 2022). To ensure isotropic expansion and eliminate any possibility of denaturation-induced deformations, we compared the same cells before and after expansion (Fig. S1A,B). We observed an intact cellular morphology with preserved organellar integrity and location at all stages of the cell cycle (Fig. S1B). As expected, post-denaturation and expansion, the organelles became more prominent compared to the pre-expanded cells (Fig. S1B). NHS-ester labelling highlighted specific organelles like mitochondria and nuclei. This experiment also enabled us to visualise the nucleolus as a region of higher protein density within the nucleus, and the SPBs as dark-stained punctate signals at the nuclear periphery and positioned away from the nucleolus (Fig. 1D).

*C. albicans* possesses a remarkable ability to switch between various morphological states, such as from unicellular yeast to hyphae, which is crucial for virulence (Sudbery et al., 2004). We, therefore, sought to assess whether *C. albicans* hyphal cells can also be expanded and whether these cells display any structural variations from the yeast form. After hyphal induction by the addition of fetal bovine serum, the cells were fixed, digested and expanded, as explained above. Pan-labelling revealed similar subcellular structures (nucleus, nucleolus, mitochondria, and SPBs) in hyphae to those seen in the budding yeast (Fig. 1E). We observed a striking difference in both the number and shape of mitochondria in hyphae compared to yeast cells (Fig. 1E). By combining NHS-ester with Hoechst 33342 staining, we were able to capture the process of nuclear migration to the germ tube, elongated nuclei, nuclei connected by a mitotic bridge and finally being segregated into two cells (Fig. 1E). We conclude that *C. albicans* can be fully expanded using U-ExM and that pan-labelling enables the identification of various subcellular structures and stages of cell division both in yeast and hyphal cells.

### Analysis of U-ExM images suggests that organellar segregation patterns during the cell cycle are evolutionarily conserved

**Fig. 2.**
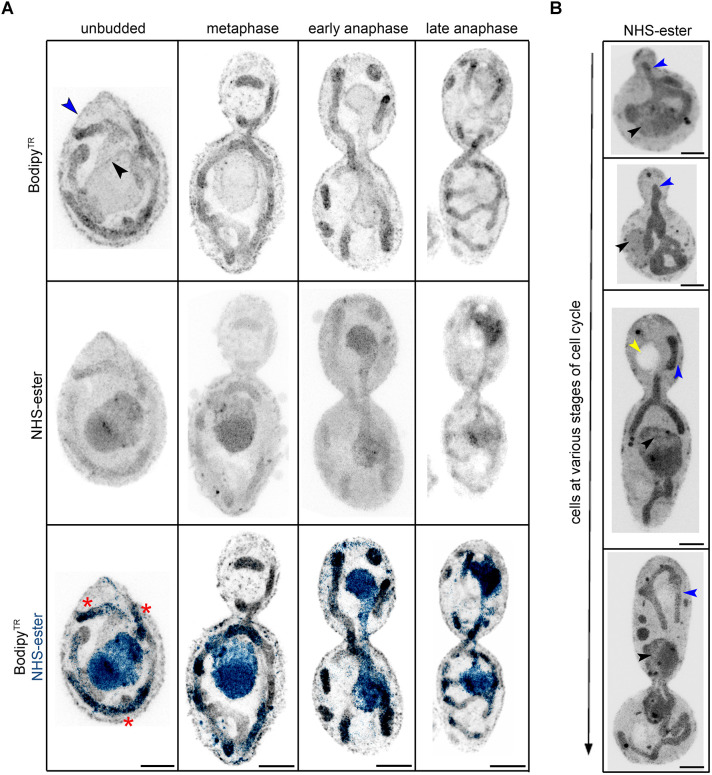
**The tubular mitochondrial network segregates into daughter cells before nuclear segregation in *C. albicans*.** (A) Maximum intensity projection of *C. albicans* cells co-stained with Bodipy^TR^ (grey) and NHS-ester (blue), during cell division. The blue and black arrowheads mark the cell and nuclear membrane, respectively. Red asterisks mark the mitochondria labelled with both Bodipy^TR^ and NHS-ester. Scale bars: 5 µm. (B) Maximum intensity projection of *C. albicans* cells stained with NHS-ester (grey), during the cell cycle displays segregation of mitochondria (blue arrowheads) before nuclear (black arrowheads) segregation between the two cells. The yellow arrowhead marks the unlabelled vacuole in the cell. Scale bars: 5 µm. All images representative of three experimental repeats. Images are from post-expansion U-ExM; scale bars have not been rescaled for the gel expansion factor.

The cell cycle-dependent morphology of the mitochondrial network plays a central role in the growth and fitness of organisms by influencing metabolism and regulating various signalling cascades (Giacomello et al., 2020). Having seen a mitochondrial-like tubular network upon pan-labelling, we assessed whether these organelles were indeed mitochondria. For this, we co-stained *C. albicans* cells with the NHS-ester 405 and Bodipy^TR^ Ceramide. Bodipy^TR^ selectively stains lipid-rich organelles like the Golgi complex and mitochondria (Adisa et al., 2003). Indeed, co-staining confirmed the dense tubular network as mitochondria (Fig. 2A). *C. albicans* displayed a tubular mitochondrial network at all stages of the cell cycle (Fig. 2A). Recently, a preferred order of organelle inheritance was shown in *S. cerevisiae*, wherein mitochondria are inherited before the migration of the nucleus into the daughter bud (Li et al., 2021). The analysis of the mitochondrial network and unstained vacuole during the cell cycle suggests that like *S. cerevisiae*, *C. albicans* cells consistently inherited both mitochondria and vacuoles before the migration of the nucleus during the cell cycle (Fig. 2B).

**Fig. 3.**
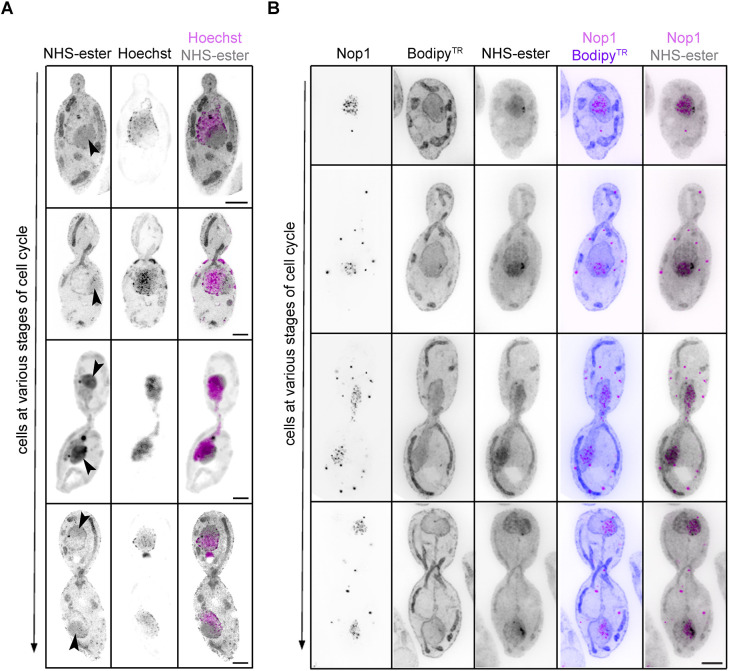
**Nucleolar segregation during cell division in *C. albicans*.** (A) Maximum intensity projection of U-ExM images of *C. albicans* cells co-stained with Hoechst 33342 (magenta) and NHS-ester (grey) during cell division. The black arrowheads mark the nucleolus. Scale bars: 5 µm. (B) Maximum intensity projection of *C. albicans* cells co-stained with NHS-ester (grey), Bodipy^TR^ (blue) and Nop1 Abs (magenta) at various stages of the cell cycle. Scale bar: 5 µm. All images representative of three experimental repeats. Images are from post-expansion U-ExM; scale bars have not been rescaled for the gel expansion factor.

The NHS-ester pan-labelling also enabled us to focus closely on nuclear structures. We found a strong NHS-ester staining within the nucleus, which corresponded to the nucleolus (Fig. 3A). This was evidenced by Hoechst 33342 staining, which is mostly excluded from the nucleolus and stains chromatin (Fig. 3A). We also noticed a darkly stained region within the nucleolus of unknown identity (Fig. S2A). *C. albicans* did not exhibit a typical crescent-shaped nucleolus during interphase (Fig. 3A) as reported for *S. cerevisiae* (Girke and Seufert, 2019). Based on the budding index and having prior knowledge of cell cycle progression in *C. albicans* (Berman, 2006; Sreekumar et al., 2021), the snapshots captured at various stages of mitosis do enable a fair idea of the organellar dynamics. We, therefore, co-stained with NHS-ester and Hoechst 33342, which helped us observe nucleolar segregation dynamics during the cell division in *C. albicans*. The nucleolus remained closely associated with the Hoechst 33342-stained chromatin mass and segregated alongside bulk chromatin (Fig. 3A). This resembles the nucleolar segregation pattern seen in *S. cerevisiae* and in hyphal-induced *C. albicans* cells (Finley and Berman, 2005; Girke and Seufert, 2019; Granot and Snyder, 1991)*.* We also validated the dark-stained region to be the nucleolus by staining for Nop1. Nop1 is a component of the small subunit processome complex and is required for the processing of pre-18s rRNA and localises to the nucleolus (Schimmang et al., 1989). Indeed, anti-Nop1 antibodies colocalised with the higher NHS ester-stained region within the nucleus during the cell cycle (Fig. 3B). Importantly, within the nucleolus, anti-Nop1 immunostaining revealed regions of higher and lower fluorescence intensities (Fig. 3B), reflecting differential intensities of Nop1. Taken together, NHS-ester pan-labelling provides an expansive view of the cellular landscape at various stages of the cell cycle in *C. albicans*.

### U-ExM provides insight into the organisation, assembly and inheritance of SPBs in *C. albicans*

**Fig. 4.**
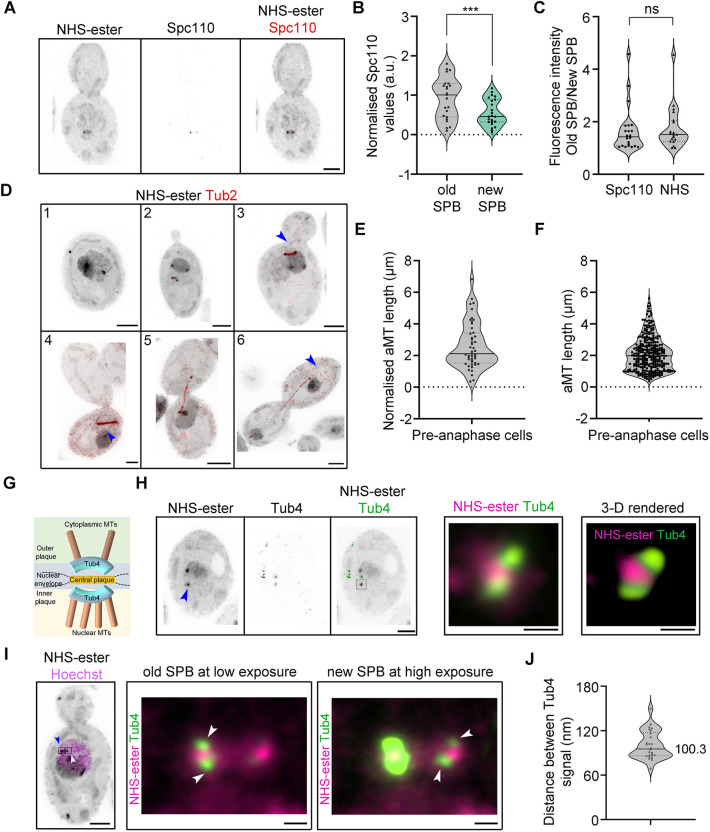
**SPB organisation in *C. albicans*.** (A) Maximum intensity projection of *C. albicans* cells, co-stained with ­NHS-ester (grey) and anti-GFP (Spc110–GFP, red). Scale bar: 5 µm. (B) The violin plot displays Spc110 signal intensities. The signal was quantified and normalised to the mean of SPB1 (old SPB). *n*=23 expanded cells analysed. ***P=0.0005 (two-tailed paired *t*-test). (C) The violin plot displays the ratio of fluorescence intensities between old and new SPB as determined by NHS-ester signal intensities. The signal was quantified and the old SPB signal was normalised to the signal from the new SPB. *n*=22 (Spc110) and 19 (NHS-ester) expanded cells analysed. ns, not significant (two-tailed unpaired *t*-test). (D) Maximum intensity projection of *C. albicans* cells co-stained with ­NHS-ester (grey) and anti-GFP (Tub2–GFP, red), at various stages of the cell cycle (1, 2, 3, 4, 5 and 6). The blue arrowheads mark the astral microtubules (aMTs). Scale bars: 5 µm. (E) A violin plot showing the distribution of aMT length at the pre-anaphase stage of the cell cycle in expanded *C. albicans* cells. *n*=47 aMTs analysed. (F) A violin plot showing the distribution of aMT length at the pre-anaphase stage of the cell cycle from live-cell of *C. albicans* tagged with Tub2–GFP. *n*=303 aMTs analysed from 11 live-cell movies. (G) Schematic showing the spatial position of Tub4 at the SPB as reported from *S. cerevisiae*. (H) A representative maximum-intensity projection of *C. albicans* cells co-stained with NHS-ester (grey) and anti-GFP (Tub4–GFP, green) in unbudded cells. Scale bar: 5 µm. Right, a magnified view of the region depicted by a black-bordered square and a 3D rendered image of the magnified region. Scale bars: 500 nm. (I) Maximum-intensity projection of *C. albicans* cells co-stained with NHS-ester (grey) and Hoechst 33342 (magenta) at the pre-anaphase stage of the cell cycle. Scale bar: 5 µm. The blue and white arrowhead represents the old and new SPBs, based on signal intensity. Right two images, Right, a magnified view of the region depicted by a black-bordered rectangle surrounding the two SPBs. The first image highlights the Tub4 arrangement (white arrowheads) at the old SPB, visible at low-intensity exposure. The second image highlights the Tub4 arrangement (white arrowheads) at the new SPB visible only at high-intensity exposure. Scale bars: 500 nm. (J) A violin plot displaying the distance between two Tub4 fluorescent signals rescaled after expansion (*N*=2 experiments, *n*=26 SPBs). Violin plots have the median (solid line) and the 1st and 3rd quartiles (dotted line) marked. All images representative of three experimental repeats. Images are from post-expansion U-ExM; scale bars have not been rescaled for the gel expansion factor.

SPBs nucleate microtubules (MTs), regulating nuclear positioning and spindle alignment during cell division. Although the role of SPBs during the cell cycle is well known for the model yeasts *S. cerevisiae* and *S. pombe*, the organisation, assembly and inheritance of SPBs are poorly understood in pathogenic fungi like *C. albicans*. Densely packed with proteins, SPBs in most species tend to be visible as a bright punctate structure in NHS-ester labelling (Shah et al., 2023; M'Saad and Bewersdorf, 2020) and also tend to be positioned away from the nucleolus in *Cryptococcus neoformans*, *Exophiala dermatitidis* and *S. cerevisiae* (Yamaguchi et al., 2010; Yamaguchi et al., 2003; Jin et al., 2000; Yang et al., 1989). In line with this, we also observed a bright punctate signal positioned away from the nucleolus and colocalising with chromatin (Hoechst 33342 staining) in *C. albicans* (Fig. 1D; Fig. S3A). To validate the identity of bright punctate structures as SPBs, we tagged Spc110, an inner plaque component of the SPB, with GFP and carried out IF staining using anti-GFP antibodies after the expansion of cells. Co-staining of Spc110 with NHS-ester confirmed these structures as SPB throughout the cell cycle (Fig. S3B). Differential staining was observed between the two SPBs both by NHS-ester labelling and Spc110–GFP IF (Fig. 4A; Fig. S3B). This asymmetry in protein density was validated by Spc110–GFP quantification, which showed the old SPB to be ∼1.8-fold brighter than the other SPB at pre-anaphase (Fig. 4B). This is in corroboration with an earlier observation in *S. cerevisiae*, where the new SPB was reported to be fainter than the old SPB (Liakopoulos et al., 2003). The ratio of old and new SPB fluorescence intensities determined by NHS-ester labelling was consistent with Spc110 IF values (Fig. 4C). This reiterates the utility of NHS-ester labelling in visualising and estimating protein density differences.

We also validated these structures to be the MT nucleation centres by immunostaining tubulin in a strain carrying Tub2–GFP (Fig. 4D)*.* U-ExM revealed the structural changes in the mitotic spindle during the cell cycle, with the spindle being compact at early cell cycle stages, evident from intense staining (stages 1–4, Fig. 4D). As the cells entered into anaphase, the mitotic spindle showed low staining due to the presence of fewer kinetochore MTs (stages 5–6, Fig. 4D), as reported for *S. cerevisiae* (Winey and O'Toole, 2001). The U-ExM images with tubulin staining also suggested variable aMTs length during pre-anaphase stages, ranging from 0.35 to 6.82 µm (mean, 2.61 µm) (Fig. 4E; Movies 1–3). Live-cell analysis of MT dynamics also showed aMT length to vary from 0.36 to 5.62 µm (mean=2.10 µm) (Fig. 4F). Our analyses show longer aMTs in *C. albicans* than in *S. cerevisiae* (mean<1.5 µm, metaphase) (Zucca et al., 2023). *C. albicans* also has free cytoplasmic MTs (cMTs) and their presence is cell cycle dependent (Finley and Berman, 2005; Lin et al., 2016). We could also stain cMTs in expanded cells of *C. albicans* (Movie 2). MT dynamics in live cells revealed that the appearance of free cMTs mirrored the depolymerisation of MTs during telophase (Fig. S4A).

The SPB structure and its duplication during the cell cycle are well-studied in two model yeasts, *S. cerevisiae* and *S. pombe* (Cavanaugh and Jaspersen, 2017). The SPB is divided into inner, central and outer plaques in *S. cerevisiae*. The central plaque anchors the outer and inner plaques, which nucleate astral and cytoplasmic MTs and the nuclear MTs, respectively. One of the applications of isotropic expansion is the decrowding of the intracellular space, which provides a way to study the effective spatial resolution of proteins (Tillberg and Chen, 2019; Wassie et al., 2019). To investigate the effective resolution of two plaques of SPB in *C. albicans*, we resorted to the γ-tubulin homolog Tub4, which is positioned on both inner and outer plaques (Fig. 4G). GFP-tagged Tub4-expressing *C. albicans* cells were expanded and probed with anti-GFP antibodies. Using Airyscan imaging, we could obtain two Tub4 fluorescence signals, representing the inner and outer plaques in unbudded cells (Fig. 4H). We were also able to detect four Tub4 dots, two from each SPB, post-SPB duplication (Fig. 4I). The more intense old SPB, evident from NHS-ester labelling, showed symmetric Tub4 signals between the inner and outer plaque. However, we observed a difference in the signal intensity between the two Tub4 signals for the less intense new SPB (Fig. 4I). We estimated the distance between the two Tub4 signals after 2D projection and found them to be separated by 100.3±5.3 nm (mean±s.e.m.) (Fig. 4J), after rescaling for the expansion factor. Altogether, using U-ExM, we could study SPB asymmetry and estimate the distance between the outer and inner plaque by resolving Tub4 fluorescent signals in *C. albicans*.

Separation of the duplicated SPBs, followed by their movement to the diametrically opposite sides of the nuclear envelope, is a prerequisite for the formation of a bipolar mitotic spindle in *S. cerevisiae* (Cavanaugh and Jaspersen, 2017). To study the SPB separation dynamics in *C. albicans*, we tagged the spindle with Tub2–GFP and SPBs with Tub4–mCherry (Fig. S5A–C). For comparison, spindle and SPBs were tagged with GFP–Tub1 and Spc42–mCherry, respectively in *S. cerevisiae* (Reza et al., 2024 preprint). We looked at the distribution of the pre-anaphase spindle length with respect to the budding index. We found that the spindle in *C. albicans* was restricted to a length of <1 µm when compared to the 1–1.5 µm spindle in *S. cerevisiae* (Fig. S5D), resulting in unequal partitioning of the 2D-projected chromatin-covered nuclear area (major and minor segments) (Fig. S5E). To validate this, we measured the proportion of chromatin covered by the minor segment (see Materials and Methods) in pre-anaphase cells. In *C. albicans*, the minor segment constituted 23% of the chromatin area, whereas in *S. cerevisiae* we observed a 32% coverage (Fig. S5F), suggesting a side-by-side arrangement of SPBs in *C. albicans* as opposed to a pole-to-pole arrangement in *S. cerevisiae*.

**Fig. 5.**
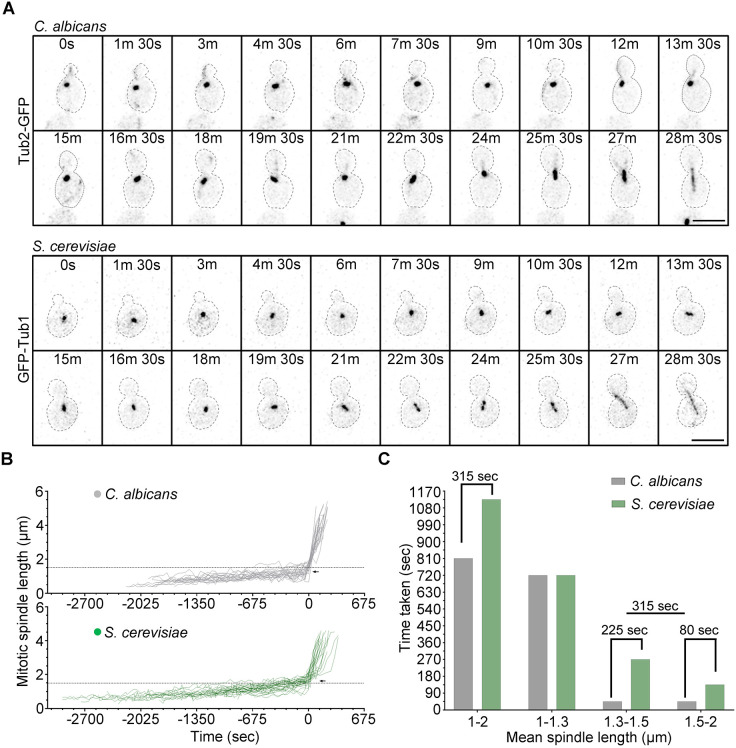
***C. albicans* displays a faster anaphase onset with a shorter pre-anaphase spindle.** (A) A montage of time-lapse images showing dynamics of the mitotic spindle, tagged with GFP, during the cell cycle in *C. albicans* (*top*) and *S. cerevisiae* (*bottom*) at 30°C. Scale bar: 5 μm. (B) Spindle length progression during cell cycle in *C. albicans* (grey) and *S. cerevisiae* (green). *n=*25 and 23 live-cell movies for *C. albicans* and *S. cerevisiae*, respectively. The horizontal line (black dotted) is drawn at 1.5 μm spindle length as a reference point. The black arrow indicates anaphase onset. The first time point corresponding to ≥2 μm spindle length is set to zero to align all the live-cell movie data. (C) Bar graph displaying the time taken (*y-*axis) for reaching maximum mean spindle length in *C. albicans* (grey) and *S. cerevisiae* (green) for different categories. The difference in time taken for each category of mean spindle length between *C. albicans* and *S. cerevisiae* is mentioned in the graph.

We further probed into the mitotic spindle dynamics during cell division by live-cell in *C. albicans* and *S. cerevisiae* (Fig. 5A). *S. cerevisiae* cells appear to transit to anaphase at a spindle length of >1.5 µm (Helsen et al., 2024; Yang et al., 1997), whereas *C. albicans* cells achieved this transition before the spindle length reached 1.5 µm (Fig. 5B). *C. albicans* displayed a shorter metaphase duration owing to the early transition from metaphase-to-anaphase at a shorter pre-anaphase spindle in comparison to *S. cerevisiae* (Fig. 5C; Fig. S5G,H), which supports our above observation of lower coverage of the chromatin in *C. albicans* (Fig. S5F). The onset of anaphase in *C. albicans* was at a shorter spindle length of 1.3 µm compared to the >1.5 µm spindle in *S. cerevisiae* (Fig. 5C; Fig. S5G,H), which explains the difference in time spent with spindles between 1 and 2 µm, as both the yeasts spend the same time with a 1–1.3 µm spindle length (Fig. 5C). Before anaphase onset, *C. albicans* displayed significantly shorter spindle as compared to *S. cerevisiae* (Fig. S5H). In summary, *C. albicans* shows an atypical arrangement of duplicated SPBs with shorter pre-anaphase spindles along with the presence of long aMTs and free cMTs, suggesting different MT dynamics and regulation compared to *S. cerevisiae*.

### U-ExM as a broadly applicable tool to investigate ultrastructure in human fungal pathogens

**Fig. 6.**
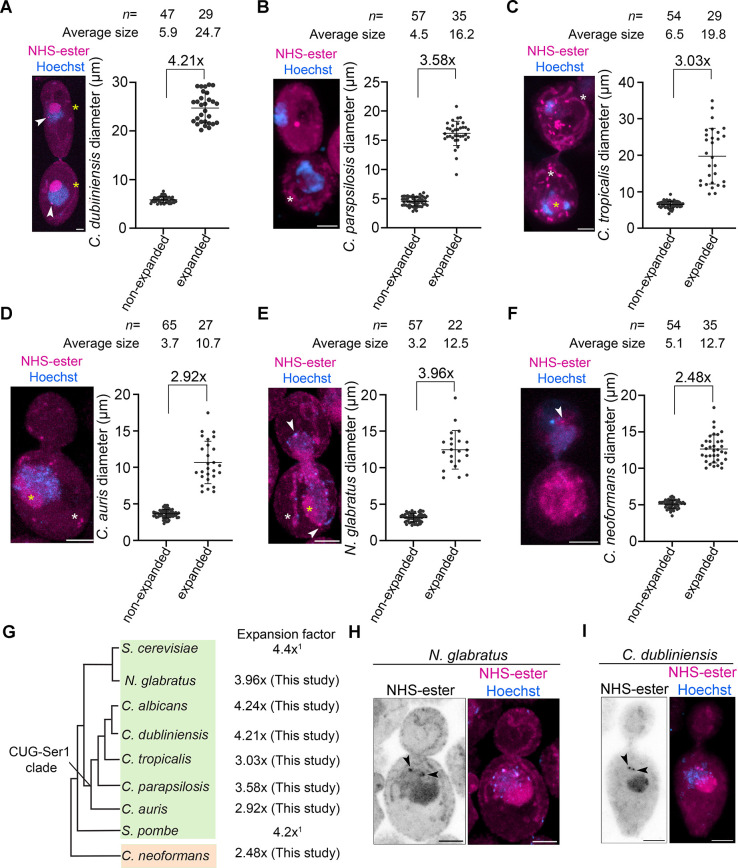
**Expansion followed by pan-labelling reveals the subcellular organisation in human fungal pathogens.** (A–F) Confocal images of human fungal pathogens post-expansion, co-stained with NHS-ester (magenta) and Hoechst (blue) and displayed as maximum intensity projection (left). Corresponding scatter plot (right) displaying the expansion factor based on measurement of the diameter of unbudded cells for these species before and after expansion. Error bars show mean±s.d. The white arrowheads represent SPBs. The nucleolus and mitochondria are labelled with yellow and white asterisks, respectively. Scale bars: 5 µm. (G) Cladogram showing the human fungal pathogens used in this study for U-ExM, Ascomycota (green) and Basidiomycota (orange). The expansion factor for these fungal pathogens is mentioned along with the well-known budding and fission yeast, *S. cerevisiae* and *S. pombe* (1, Hinterndorfer et al., 2022), respectively. (H) Representative confocal image of *N. glabratus* post-expansion, pan-labelled with NHS-ester (magenta) and Hoechst 33342 (blue) and displayed as maximum intensity projection. The black arrowheads show sideward SPB arrangements (*n=*19, budding index= 0.33–0.69). (I) Representative confocal image of *C. dubliniensis* post-expansion, pan-labelled with NHS-ester (magenta) and Hoechst 33342 (blue) and displayed as maximum intensity projection. The black arrowheads show sideward SPB arrangements (*n=*12, budding index= 0.47–0.75). Scale bars: 5 µm. Scale bars have not been rescaled for the gel expansion factor.

We extended the optimised U-ExM protocol of *C. albicans* to several human fungal pathogens belonging to diverged fungal lineages: the CUG-Ser1 clade and the WGD lineage of Ascomycota, and a species that belongs to Basidiomycota. Modifications in the *C. albicans* ExM protocol for each of these species are included in the Materials and Methods. For most of these species, increasing the incubation time from 45 min to 60 min for cell wall digestion facilitated near-complete expansion, except for *Candida auris*, *Candida tropicalis* and *C. neoformans*. *C. neoformans* required an additional Triton X-100 treatment before the cell wall digestion for increased efficiency. We demonstrate that *Candida dubliniensis* and *Candida parapsilosis,* two CUG clade species, related to *C. albicans*, could be expanded 4.21- and 3.58-fold, respectively (Fig. 6A,B). *C. tropicalis* and *C. auris*, also belonging to the CUG-Ser1 clade, showed an expansion factor of 3.03 and 2.92, respectively (Fig. 6C,D). Although the ascomycete, *Nakaseomyces glabratus*, which belongs to the WGD clade could be expanded by 3.96-fold, the basidiomycete *C. neoformans* showed an expansion factor of 2.48-fold only (Fig. 6E,F). We could observe various subcellular structures like nuclei, nucleolus, SPBs, vacuoles, mitotic bridge and mitochondria in the expanded human fungal pathogens (Fig. S6A). Together, we demonstrate a successful optimisation of the expansion of *C. dubliniensis*, *C. parapsilosis* and *N. glabratus* (Fig. 6G). Differences in several subcellular structures were evident across the species. Mitochondria in *C. dubliniensis* and *N. glabratus* were tubular, whereas *C. tropicalis* displayed both tubular and fragmented mitochondrial networks (Fig. 6A–F). Having seen a side-by-side arrangement of SPBs in *C. albicans*, we were curious to know the SPB arrangements in *C. dubliniensis* and *N. glabratus.* Remarkably, we observed a side-by-side arrangement of SPBs in both these species (Fig. 6H,I). Thus, U-ExM followed by pan-labelling identified the conservation in side-by-side SPB arrangements in these two species.

## DISCUSSION

In this work, we optimised the U-ExM protocol in the human fungal pathogen *C. albicans*, a model system to study cell division and host–pathogen interactions. Expansion together with pan-labelling of the proteome facilitated the monitoring of organellar segregation dynamics via snapshots of cell division events in *C. albicans*. With a 4-fold expansion, we could successfully resolve the inner and outer plaques of the SPBs. We extended this protocol to expand its application to study the cell biology of some of the non-model medically relevant human fungal pathogens.

Dual dye staining revealed the tubular mitochondria being inherited before the nucleus into the daughter cell in *C. albicans*, whereas the nucleolus moves in conjunction with chromatin during the cell cycle, as evident from immunostaining of Nop1. This highlights the compatibility of U-ExM with dual dye and immunostaining. Unlike the well-studied model organism *S. cerevisiae*, a non-crescent-shaped nucleolus was observed with seemingly large occupancy in the *C. albicans* nucleus. The nucleolus is known to be important for ribosome biogenesis and regulation, and a change in size, number and structure of nucleolus is often associated with various cellular metabolic states (McCann and Baserga, 2014). Although nutrient restriction leads to a reduction in size (Matos-Perdomo and Machín, 2019), metabolically active cells have an enlarged nucleolus (Weeks et al., 2019). Whether a difference in the lifestyle between pathogenic (*C. albicans*) and non-pathogenic (*S. cerevisiae*) organisms relates to a differential nucleolar shape and occupancy is an area for further research.

U-ExM is an excellent tool for studying differential protein occupancy, which becomes pronounced due to a dilution of fluorescence intensity upon expansion (Götz et al., 2020). This was evident for the SPB proteins Spc110 and Tub4, which revealed a difference in protein density between the old and new SPBs. The asymmetric Tub4 distribution was also apparent between the inner and outer plaque at the newly formed SPBs. Unlike *S. cerevisiae* (Geymonat et al., 2020), *C. albicans* does not show any noticeable asymmetric distribution of Tub4 between the two plaques during interphase and in the old SPBs post-duplication by U-ExM. A shorter Tub4-to-Tub4 distance in *C. albicans* further hints towards an SPB organisation distinct from *S. cerevisiae* (Burns et al., 2015; Byers and Goetsch, 1974; Hinterndorfer et al., 2022). *C. albicans* displaying a side-by-side SPB arrangement in pre-anaphase unlike the pole-to-pole arrangement seen in *S. cerevisiae*, further hints towards a difference in SPB separation events. Thus, we could obtain a pronounced view of SPB organisation and critically analyse the differences in SPB features in *C. albicans* using U-ExM, overcoming the limitations of conventional microscopy.

In this study, we show that the U-ExM protocol can be applied to six other human fungal pathogens, belonging to Ascomycota and Basidiomycota fungal phyla. The cell wall of *C. albicans* is known to have more β-1,6-glucans than that of *S. cerevisiae* (Brown and Gordon, 2005), which is reflected in the timing of cell wall digestion, with *C. albicans* requiring a longer time for complete cell wall digestion than *S. cerevisiae* (Hinterndorfer et al., 2022). In our study, a 4-fold expansion could not be achieved for *C. auris*, *C. tropicalis* and *C. neoformans*. Composed of α-1,3-glucan, β-1,3 and β-1,6-glucan, chitin, chitosan, mannoproteins and GPI-anchored proteins (Garcia-Rubio et al., 2020), the two-layered cell wall of *C. neoformans* is further surrounded by an exopolysaccharide capsule (Garcia-Rubio et al., 2020). This vastly differs from the cell wall properties of *C. albicans* and related *Candida* species, explaining the reduction in the expansion factor observed. By contrast, the *C. auris* and *C. tropicalis* isolates used in this study are resistant and tolerant to fluconazole (National Culture Collection of Pathogenic Fungi; https://nccpf.in/), respectively, which is implicated with increased levels of cell wall chitin (Shahi et al., 2022). This could be the likely reason for the inefficiency in achieving a 4-fold expansion despite *C. auris* and *C. tropicalis* being related species to *C. albicans*.

The side-by-side arrangement of SPBs observed in this study for *C. albicans* was also reflected by the NHS-labelled SPBs in *C. dubliniensis* and *N. glabratus*. This demonstrates the importance of U-ExM in the study of various cell biological processes in the absence or relative ease of techniques available for native tagging, live-cell microscopy and standardised transformation protocols for various non-model organisms. Our analysis suggests that despite being a member of the WGD clade, *N. glabratus* SPBs may not follow segregation dynamics akin to *S. cerevisiae*. This divergence in SPB arrangements between WGD species calls for further studies as molecular details regarding the role of SPBs in asymmetric cell division, inheritance and evolution in fungal pathogens are still lacking.

## MATERIALS AND METHODS

### Yeast strains and culture

All the strains and primers used in this study are specified in Tables S1 and S2, respectively.

### Construction of a strain expressing Tub2–GFP and Tub4–mCherry

The 3′ non-coding region of Tub2 after the stop codon was amplified from *C. albicans* (SN148) genomic DNA using primers RG040/RG041 and cloned into the HindIII and KpnI sites of pBSGFP-His vector (Chatterjee et al., 2016). A ∼504 bp Tub2 homology region without the stop codon was PCR amplified using primers RG038 and RG039 and cloned at SacII and XbaI sites in the above-generated plasmid (pRG012) to give pRG013. The plasmids were confirmed by restriction analyses. The plasmid was transformed by insert release of the tagging cassette with SacII and KpnI. The *C. albicans* transformants were confirmed by PCR using primers RG042 and PB17 and checked for any growth difference with respect to the parent control.

### Reagents used in the study

The following primary antibodies were used in this study: anti-Nop1 [anti-fibrillarin antibody [38F3], nucleolar marker (ab4566)] used at 1:500 and anti-GFP (mouse) (Roche, 11814460001) used at 1:500. The following secondary antibodies were used in this study: Alexa Fluor 488-conjugated goat anti-mouse IgG (Invitrogen, A11001). The secondary antibodies were used at 1:500 dilution. The following NHS-ester dyes were used in the present study: Dylight^TM^ 405 NHS-ester (Thermo Fisher Scientific, 46400), Dylight^TM^ 594 NHS-ester (Thermo Fisher Scientific, 46412), and Alexa Fluor^TM^ 647 carboxylic acid, succinimidyl ester (Thermo Fisher Scientific, A20006), all used at 1:500. Bodipy^TR^ Ceramide (Invitrogen, D7540), formaldehyde (Thermo Fisher Scientific, 24008), acrylamide (Merck, A4058), N,N′-methylenebisacrylamide (Merck, M1533), Sodium acrylate (Merck, 408220-256), ammonium persulphate (APS) (HiMedia, MB003), TEMED (Merck, T7024), Hoechst 33342 (Sigma, B2261).

### Yeast culture, fixation and cell wall digestion

Briefly, log-phase cells [at ∼1 optical density at 600 nm (OD_600_) unit] were first fixed with 3.7% formaldehyde for 15 min at 30°C with intermittent shaking. 1 OD_600_ equivalent cells were taken forward for subsequent cell wall digestion. For *C. albicans*, C*. tropicalis*, *N. glabratus*, *C. dubliniensis* and *C. parapsilosis*, cells were washed twice with PEM buffer (100 mM PIPES, 1 mM EGTA, 1 mM MgSO_4_, pH 9.0) and pelleted at 2350 ***g*** for 5 min or 15 min for *C. auris*, and washed once with PEM-S (1.2 M sorbitol in PEM buffer) and pelleted at 2350 ***g*** for 5 min, or 15 min for *C. auris*. The fixed cells were resuspended in 100 µl of PEM-S buffer and enzymatically digested with a final concentration of 2.5 mg ml^−1^ Zymolyase 20T (MP, 32092) at 30°C for 45 min in the case of *C. albicans*, *C. dubliniensis*, and C*. tropicalis* and 1 h in case of *C. auris*, *C. parapsilosis* and *N. glabratus*. Cells were washed once with PEM-S buffer and pelleted at 2350 ***g*** for 5 min, or 15 min for *C. auris*, and the resuspended cells were proceeded for anchoring.

For *C. neoformans*, 1 OD_600_ equivalent cells were washed with PEM and PEM-S buffer as above. Cells were then resuspended in 500 µl of PEM-S buffer with 0.2% Triton X-100 and incubated at 30°C for 30 min at 100 rpm. This was followed by digestion using 25 mg of Lysing enzyme (Sigma, L1412) dissolved in 500 µl of PEM-S buffer and incubated at 30°C for 6 h with rotation at 100 rpm. Digested cells were taken forward for anchoring.

### Yeast-to-hyphal induction

Briefly, log-phase *C. albicans* cells (∼1 OD_600_) were added to pre-warmed media (at 37°C) containing 9 ml of YPD+uridine (10 µg ml^−1^) and 1 ml of fetal bovine serum (Thermo Fisher Scientific, 10270106). The cells were grown at 37°C for 90 min with rotation at 180 rpm for the induction of germ tube formation. The cells were fixed and proceessed for U-ExM as described above. Post-germ tube formation, cells were pelleted down at 2350 ***g*** for 15 min for every step involving centrifugation.

### U-ExM

U-ExM was performed as previously described (Hinterndorfer et al., 2022), with a few modifications. The digested cells were kept for anchoring in acrylamide (AA) and formaldehyde (FA) (1% AA and 0.7% FA diluted in 1× PBS) overnight at 37°C, with rotation at 12 rpm on a Rotaspin. The next morning, a 6 mm coverslip was coated with poly-L-lysine (Sigma, P8920) for 1 h at room temperature. The anchored cells were then allowed to attach to the poly-L-lysine coated coverslip for 1 h. Gelation was performed on ice using a cocktail of monomer solution [19% (w/v) sodium acrylate, 10% (v/v) acrylamide, 0.1% (v/v) N,N′-methylenebisacrylamide in PBS], TEMED (0.5% v/v) and APS (0.5% v/v). The cells were incubated for 10 min on ice. The gel was kept for polymerisation for 1 h at 37°C in a moist chamber. Next, the gel was transferred to 1 ml denaturation buffer (50 mM Tris-HCl pH 9.0, 200 mM NaCl, 200 mM SDS, pH to 9.0) and incubated at 95°C for 1 h 30 min at 300 rpm. After denaturation, the gel was expanded with three subsequent washes with water for 15 min each. The gel diameter was measured after expansion to determine the expansion factor. The gels expanded in the range of 3.7–4.4-fold. The gel was shrunk with three washes of 1× PBS for 10 min each. Pan-labelling for U-ExM was undertaken using Dylight^TM^ 405 NHS-ester, DyLight^TM^ 594 NHS-ester and Alexa Fluor^TM^ 647 carboxylic acid in 1× PBS overnight at 4°C.

For imaging the same pre- and post-expanded *C. albicans*, the digested cells were first kept for anchoring in AA and FA (1% AA, 0.7% FA diluted in 1× PBS) with NHS-ester and DyLight^TM^ 594 at 1:500 dilution and incubated overnight at 37°C, with rotation at 12 rpm on a Rotaspin. The anchored cells were kept for polymerisation for 1 h at 37°C in a moist chamber. The pre-expanded cells were imaged. After imaging, the gel was shrunk with three washes of 1× PBS for 10 min each. The gel was kept for denaturation at 95°C for 1 h 30 min. After denaturation, the gel was expanded with three subsequent washes with water, 15 min each. The gel was shrunk with three washes of 1× PBS for 10 min each and kept for pan-labelling using DyLight^TM^ 594 NHS-ester in 1× PBS overnight at 4°C. The next morning, the gel was expanded again with three subsequent washes with water, 15 min each. The expanded gel was used to image the same cells.

### Immunofluorescence staining

For Nop1 and GFP immunostaining, the gel was stained using anti-Nop1 and anti-GFP as the primary antibody at 1:500 and incubated overnight at 4°C. The gel was washed three times with PBS with 0.1% Tween 20 for 30 min at room temperature. The gel was then incubated with goat anti-mouse-IgG coupled to Alexa Fluor 488 secondary antibody at 1:500 and incubated at 37°C for 3 h in the dark. The antibody dilutions were prepared in 3% BSA in 1× PBS with 0.1% Tween 20. The gel was washed three times with PBS with 0.1% Tween 20 for 30 min at room temperature. The gel was expanded with three subsequent washes with water before imaging.

### Bodipy^TR^ Ceramide staining

To label the mitochondria, the gel after fixation and digestion was co-stained with Dylight^TM^ 405 NHS-ester and Bodipy^TR^ Ceramide at 1:500 dilution and incubated in 1× PBS overnight at room temperature. The next day, the gel was expanded with three subsequent washes with water for 15 min each. The expanded gel was used to image the cells.

### Sample mounting and imaging

For microscopy, poly-L-lysine coated Ibidi chamber slides (2-well, Ibidi 80287) or MatTek glass bottom dishes (P35G-0-14-C) or Cellvis (2-chambered coverglass system, C2-1.5H-N) were used. Gels were cut to an appropriate size to fit the glass bottom chambers and were overlaid with water to prevent drying or any shrinkage during imaging. The gels were imaged using the Zeiss LSM980 Airyfast confocal microscope using a Plan-Apochromat 63×/1.4 Oil DIC M2pb7 objective or LSM880 Airyfast confocal microscope using a Plan-Apochromat 63×/1.4 Oil DIC M27 or 100×/1.4 Oil DIC M27 objectives or Nikon CSU-W1 SORA using a SR P-Apochromat IR AC 60× WI/1.27 objective with 4× digital zoom.

For Figs 1E, 4A–H, 6A–I and Fig. S3B the gels were imaged with a Zeiss LSM880 AiryFast confocal microscope using a 63× oil-immersion objective (NA 1.4) at a step size of 0.3 µm.

Fig. S1 was imaged using an Andor BC43 Spinning disk microscope using a 60×/1.42 NA oil objective.

For U-ExM images, scale bars have not been rescaled for the gel expansion factor.

### Quantification of Tub4 distance in expanded *C. albicans* cells

The Tub4 distance between the inner and outer plaque of the SPB was quantified as the distance between the maximum intensities of both the signals which corresponded to the two plaques. The Tub4-to-Tub4 distance was normalized with the expansion factor.

### Quantification of SPB-to-SPB distance and nuclear partitioning by the spindle

The budding index for non-expanded cells was calculated by measuring the ratio of the diameter of the daughter and mother bud. The SPB-to-SPB distance was measured as the distance between the centre of two Tub4–mCherry (*C. albicans*) and Spc42–mCherry (*S. cerevisiae*) signals. Both budding indices and SPB-to-SPB distances were calculated using a straight-line selection tool from Fiji software. SPB-to-SPB distance and the budding index were calculated for cells having two SPB signals, thus excluding the unbudded cells and small-budded cells with single SPB puncta. To understand the proximal arrangement of SPBs on the nucleus, the Hoechst-stained nucleus was divided into two segments by a straight line covering the spindle and passing through the SPBs. The area of the minor segment of the Hoechst 33342-stained region and the whole region was calculated using the Freehand selection tool of Fiji software (the researcher was aware of the experimental conditions) and the percentage of area covered by the minor segment was used as data points.

### Live-cell imaging

*S. cerevisiae* cells carrying GFP–Tub1 (spindle) and Spc42–mCherry (SPBs), and *C. albicans* having Tub2–GFP (spindle) and Tub4–mCherry (SPBs) were grown overnight in complete medium (CM) composed of yeast nitrogen base (0.17%), ammonium sulphate (0.5%), dextrose (2%), and amino acids (10 mg/ml), re-inoculated in CM at 0.2 OD_600_ and grown for 2-3 h. The cells were kept for adherence onto the Concanavalin A-treated (Catalogue no. C2010, Sigma-Aldrich) glass-bottom dishes and incubated at 30°C for 10 min. Live-cell imaging was performed on an inverted confocal microscope (ZEISS, LSM880) equipped with a temperature-controlled chamber (Pecon incubator, XL multiSL), a Plan Apochromat 63× NA oil 1.4 objective and GaAsP photodetectors. For time-lapse microscopy related to Fig. 5, images were captured at a 45-s interval with 1% and 2% intensity exposure for *C. albicans* and *S. cerevisiae*, respectively with 0.5 µm *Z*-steps at 512×512 frame size using 488 nm for the GFP excitation wavelength.

For time-lapse microscopy related to Fig. S4, images were captured at a 2 min interval with 2% and 4% intensity exposure with 0.5 µm *Z*-steps using 488 nm and 587 nm for GFP and mCherry, respectively. All the images displayed were maximum intensity projections of images for each time made using ImageJ.

### Spc110 and NHS-ester quantification and aMT length estimation

For Spc110 levels quantification, Spc110 signals were measured from the in-focus *Z*-plane displaying the most intense signal at the pre-anaphase stage. The background signal was measured from a neighbouring region from the same plane of equal area and was subtracted from the measured Spc110 intensity. The values were normalised for both the SPBs to the SPB1 (old SPB) average intensity and plotted using GraphPad Prism 8.4.0. A similar approach was used for NHS-ester labelled SPB quantification. The fluorescence signals from old SPBs were normalised to the new SPB signals and expressed as a ratio.

The aMT length was measured after maximum intensity projection of the images using either a straight line or a freehand tool in Fiji software. The aMT length was normalised with respect to the expansion factor.

### Statistical analysis

Statistical analysis was done using GraphPad Prism 8.4.0 software. A two-tailed unpaired or paired *t*-test Student's was used.

## Supplementary Material



10.1242/joces.262046_sup1 Supplementary information
