# Supplementary material for: Expansion microscopy reveals characteristic ultrastructural features of pathogenic budding yeast species
Source: J Cell Sci. 2024 Sep 9;137(20):jcs262046. doi: 10.1242/jcs.262046 (PMC11423813; doi:10.1242/jcs.262046)
Supplement: Supplementary information [file joces-137-262046-s1.pdf]

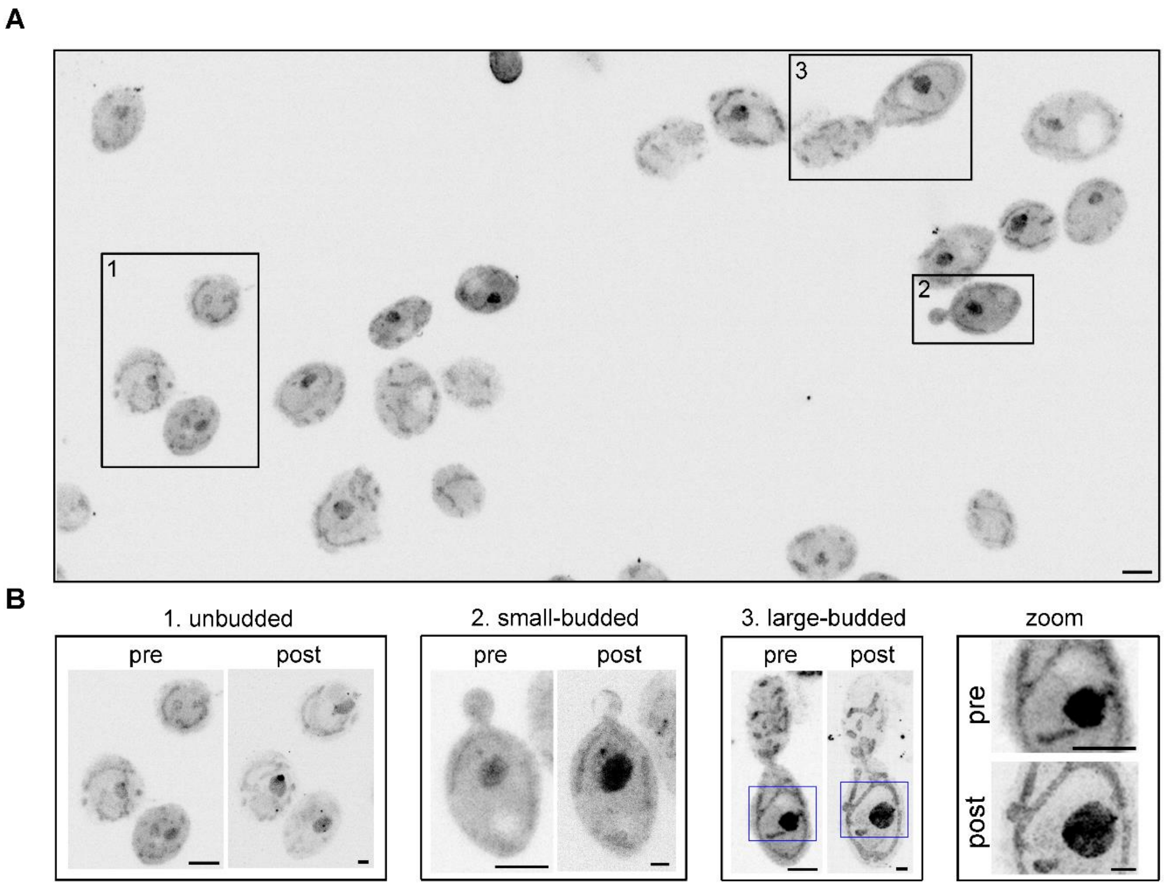

Fig. S1. Assessment of isotropic expansion of *Candida albicans* cells before and after expansion. (A) Pre-expansion: Field view of cell wall digested *C. albicans* cells embedded in gel and stained with NHS-ester (grey). Cells compared post-expansion are labelled 1,2, and 3. Scale bar 5 µm. (B) The same *C. albicans* cells were imaged pre- and post-expansion using the same microscope and objective. Images represent cells at various stages of the cell cycle. Blue insets, magnified regions showing the mitochondrial structure at pre- and post-expansion stages. Scale bar 5 µm.

Journal of Cell Science • Supplementary information

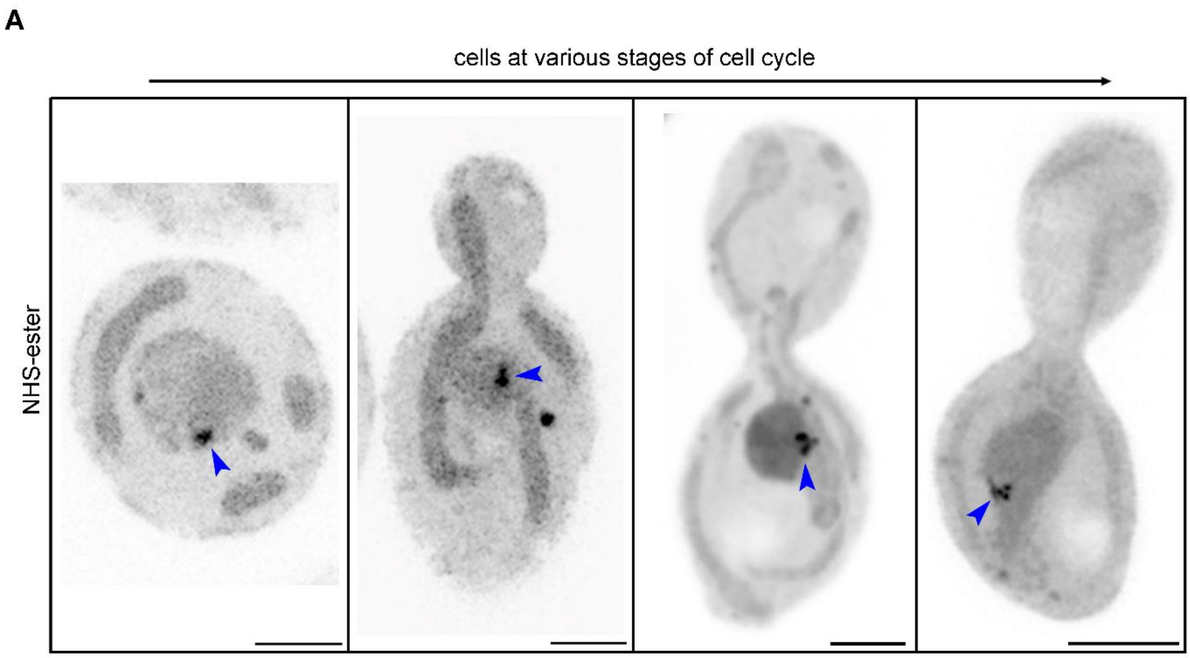

Fig. S2. U-ExM reveals a darkly stained region within the nucleolus. (A) Maximum intensity projection of *C. albicans* cells stained with NHS-ester showing the darkly stained region (blue arrowheads) within the nucleolus. Scale bar 5 µm.

Journal of Cell Science • Supplementary information

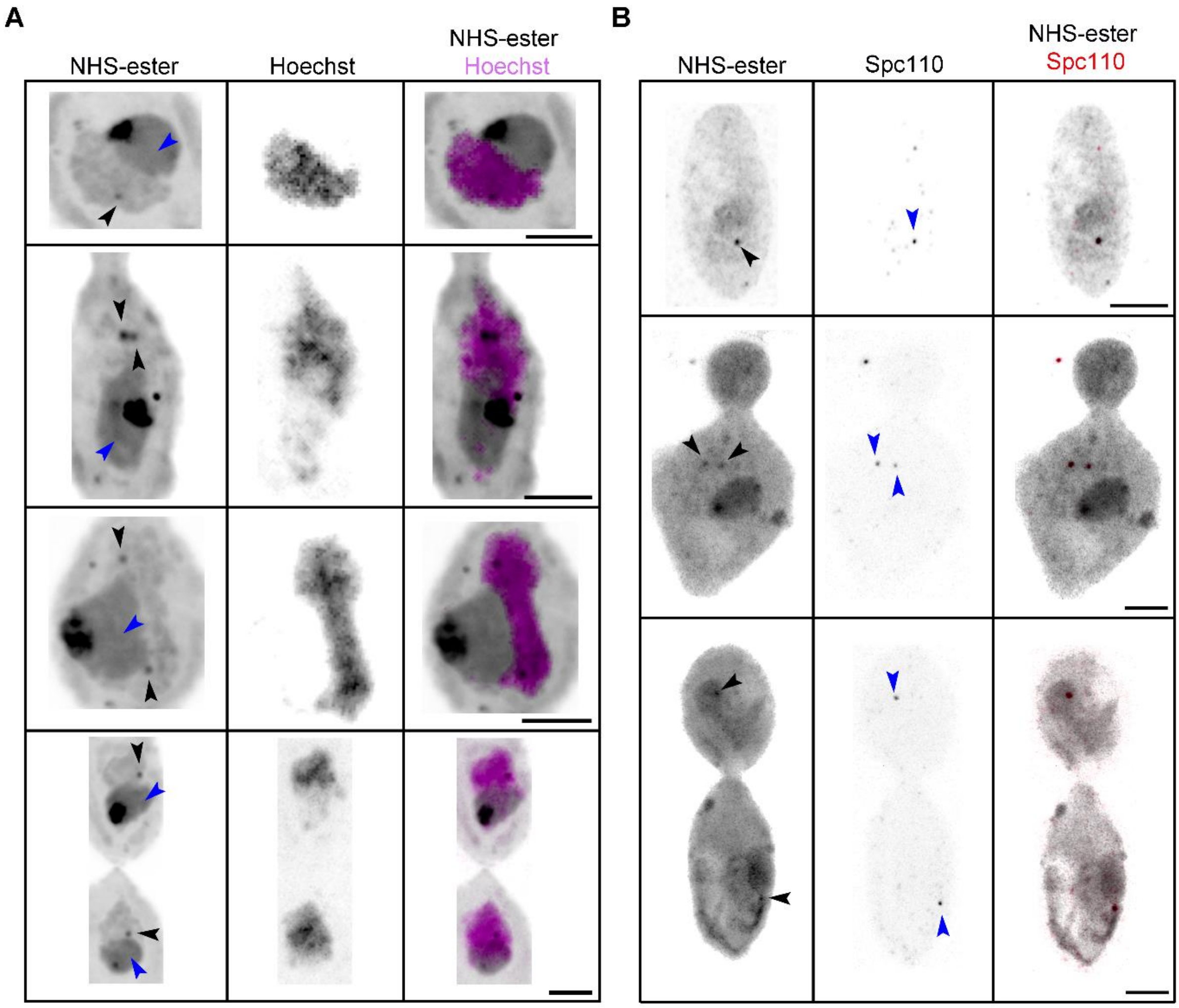

Fig. S3. SPB positioning during cell division in *C. albicans*. (A) Zoomed image showing the nucleus, co-stained with NHS-ester (grey) and Hoechst (magenta) during the cell cycle. The black arrowheads mark the position of the spindle pole bodies (SPBs) away from the nucleolus (blue arrowheads). Scale bar 5 µm. (B) Maximum intensity projection of *C. albicans* cells co-stained with NHS-ester (grey) and anti-GFP (Spc110-GFP, red) through the cell cycle. The black and blue arrowheads mark the position of the SPBs stained with NHS and the position of the SPBs stained with anti-GFP, respectively. Scale bar 5 µm.

Journal of Cell Science • Supplementary information

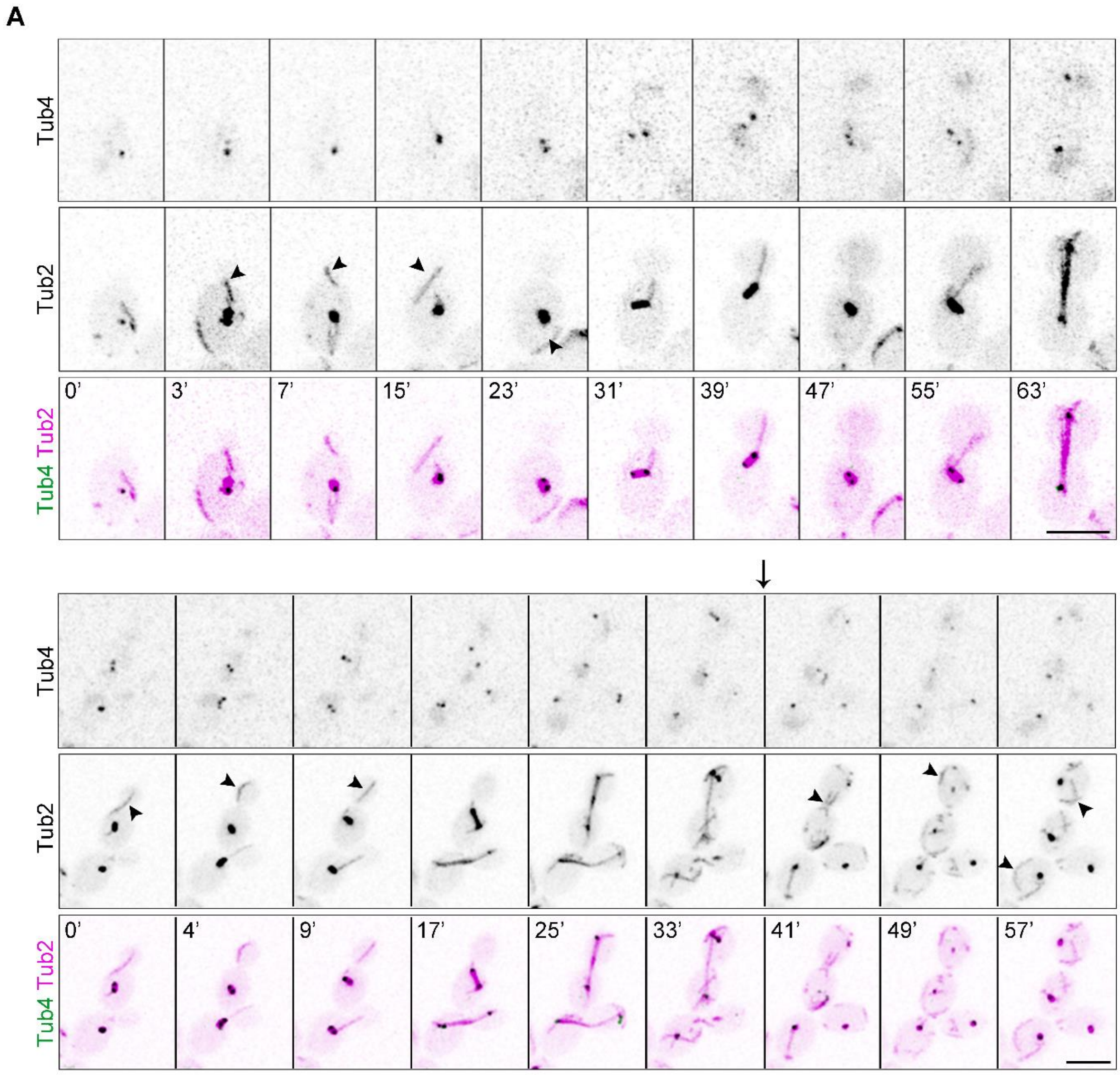

Fig. S4. Microtubule dynamics during cell division in *C. albicans*. (A) Time-lapse images showing dynamics of the microtubule (Tub2-GFP) and SPBs (Tub4-mCherry) during the cell cycle till anaphase onset (*top*) and till completion of cytokinesis (*bottom*). The black arrowheads represent free cMTs in the cytosol. The arrow marks the onset of telophase (characterised by the disassembly of the mitotic spindle). Scale bar 5 µm.

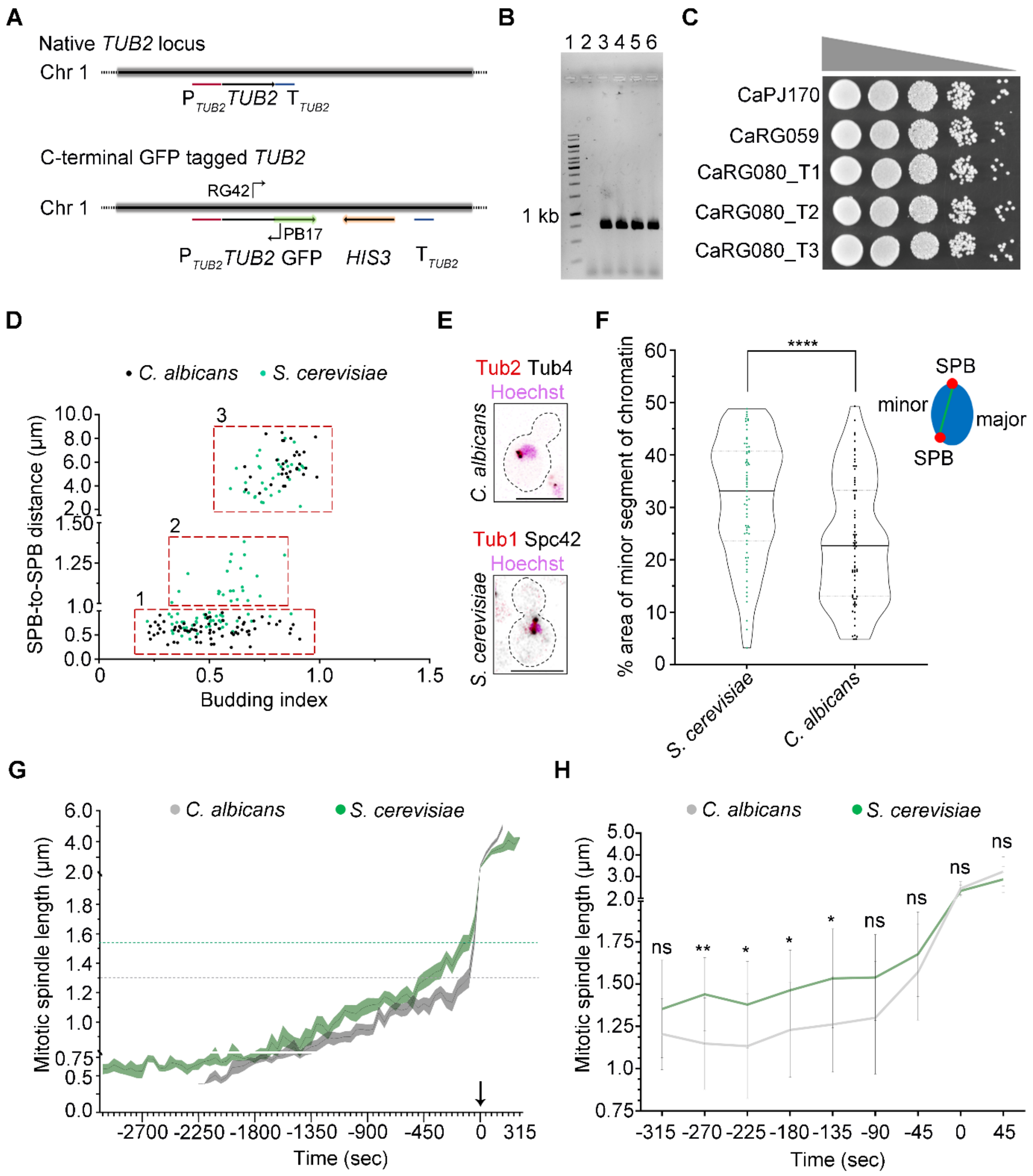

Fig. S5. SPB positioning during cell division in *C. albicans*. (A) A schematic representation showing *TUB2* tagging with GFP at the C-terminus at the native locus. Primer pairs RG42/ PB17 (~690 bp) were used to confirm the integration of the tagging cassette at the native locus. (B) A 1% agarose gel displaying the screening of the transformants by PCR using primer pairs RG42/PB17. Lane 1: 1 kb DNA ladder, lane 2: parent untagged strain (CaPJ170), lane 3: parent tagged with Tub2-GFP (CaRG059), lane 4-6: parent tagged with Tub2-GFP Tub4-mCherry (CaRG080_T1-T3). (C) Overnight grown *C. albicans* cells (CaPJ170, CaRG059, and CaRG080_T1-T3) were 10-fold serially diluted, and spotted on YPD+uridine.

Journal of Cell Science • Supplementary information

Plates were photographed after incubation at 30°C for 30 h. (D) Scatter plot displaying SPB-to-SPB distance with respect to the budding index in *C. albicans* (black) and *S. cerevisiae* (green). SPB-to-SPB distance above 2 µm represents an anaphase spindle. The red boxes represent three prominent clusters obtained in *S. cerevisiae*. *n*> 100 cells. (E) Maximum intensity projection of *S. cerevisiae*, tagged with GFP-Tub1 and Spc42-mCherry, and *C. albicans* tagged with Tub2-GFP and Tub4-mCherry and co-stained with Hoechst during the pre-anaphase stage. Scale bar 5 µm. (F) Cartoon showing the mitotic spindle (green) between the two SPBs (red) dividing the Hoechst-stained chromatin (blue) area into two unequal segments (minor and major) as visually observed in a 2D-projected image. Violin plot showing percent area covered by the minor segment of Hoechst-stained chromatin in *C. albicans* and *S. cerevisiae*. *n*> 70 cells. Statistical analysis was done by Unpaired *t*-test with Welch's correction (****p<0.0001). (G) A line graph displaying the mitotic spindle length distribution during cell cycle progression in *C. albicans* (grey) and *S. cerevisiae* (green). Anaphase onset is marked by horizontal lines, grey (*C. albicans*) and green (*S. cerevisiae*). *n*=25 and 23 live-cell movies for *C. albicans* and *S. cerevisiae*, respectively. The first time point corresponding to >=2 µm spindle length (black arrow) was used as a reference point and is set to zero to align all the live-cell movie data. The plot represents mean (black line) ±s.e.m. (H) Representation of spindle length before and after 2 µm spindle in *C. albicans* (grey) and *S. cerevisiae* (green). Zero time point corresponds to >=2 µm spindle. The lines represent mean from 25 and 23 live-cell movies for *C. albicans* and *S. cerevisiae*, respectively. Statistical analysis was done using Two-way ANOVA followed by Sidak's multiple comparisons test (**p=0.0014, *p=0.0383/0/0269/0.0186). Error bars show mean± s.d.

Journal of Cell Science • Supplementary information

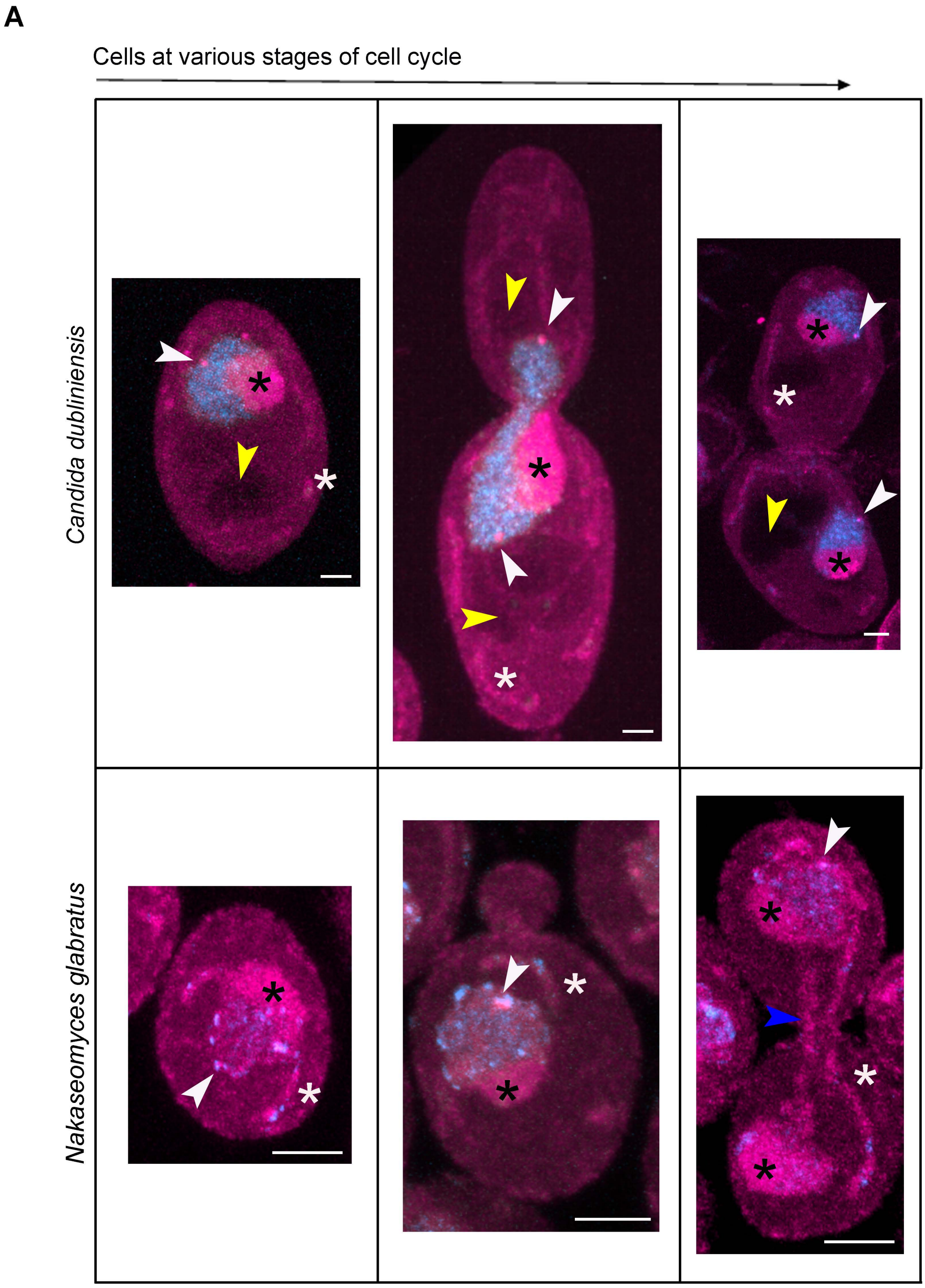

Fig. S6. Pan-labelling of proteome displays sub-cellular organisation in expanded pathogenic yeast cells. (A) Representative images of *C. dubliniensis* and *N. glabratus* co-stained with NHS-ester (magenta) and Hoechst (blue) showing sub-cellular organisation at various stages of the cell cycle. The yellow, white, and blue arrowheads represent vacuole, SPBs, and mitotic bridge respectively. The white and black asterisks mark mitochondria and nucleolus, respectively. Scale bar 5 μm.

Journal of Cell Science • Supplementary information

Table S1. List of strains used in this study.

S. No.	Strains	Genotype	Source
1.	*C. albicans* CaPJ121 (*CSA6-mCherry* in *SPC110-GFP*)	CaPJ118 *SPC110/SPC110-GFP::HIS1*	(Jaitly et al., 2022)
2.	*C. albicans* CaPJ170	SN148 *ADH1/adh1::PTDH3-cartTA SAT1 RPS1/RPS1::PTET-GtwB-URA3*	(Jaitly et al., 2022)
3.	*C. albicans* CaPJ171	CaPJ170 *TUB4/TUB4-GFP::HIS1*	(Jaitly et al., 2022)
4.	*C. albicans* CaPJ172	CaPJ170 *TUB4/TUB4-GFP::HIS1 TUB1/TUB1-mCherry::ARG4*	(Jaitly et al., 2022)
5.	*C. albicans* CaRG059	CaPJ170 *TUB2/TUB2-GFP::HIS1*	This study
6.	*C. albicans* CaRG080	CaPJ170 *TUB2/TUB2-GFP::HIS1 TUB4/TUB4-mCherry::ARG4*	This study
7.	*Nakaseomyces glabratus* CBS138		https://wi.knaw.nl/fungal_table

Journal of Cell Science • Supplementary information

**8.**	*Candida dubliniensis* CdUM4B	*ura3Δ1::FRT/ ura3Δ2::FRT*	(Staib et al., 2001)
**9.**	*Candida parapsilosis* NRRL Y-8312		https://nrrl.ncaur.usda.gov/cgi-bin/usda/fungi/report.html?nrrlcodes=Y%2d831
**10.**	*Candida auris* NCCPF470145		National Culture Collection of Pathogenic Fungi (nccpf.in)
**11.**	*Candida tropicalis* CtKS200	*ura3::FRT/ura3::FRT his1::FRT/his1::FRT arg4::FRT/arg4::FRT MIF2/MIF2-GFP (HIS1)*	(Chatterjee et al., 2016)
**12.**	*Cryptococcus neofromans* CNV114	*MAT***a** *GFP-H4::NAT mCherry-CSE4::NEO*	(Kozubowski et al., 2013)
**13.**	MHR35	*MAT***a** *his3Δ1 leu2Δ0 met15Δ0 ura3Δ0::URA3* GFP-*TUB1* Spc42-mCherry*::KanMX4*	(Reza et al., 2022)

Journal of Cell Science • Supplementary information

Table S2. List of primers used in this study.

S. No.	Primer name	Sequence 5' to 3'	Purpose
1.	RG040	TCCAAGCTTAAGCTGATAAATGCTACAATAT	For amplification of 3' coding region of *TUB2*
2.	RG041	ATGGTACCGTGGTAAAGAATAGGACATCG	
3.	RG038	ATCCGCGGAGATCAGTCACCGTCCCAG	For amplification of *TUB2* ORF prior to the stop codon
4.	RG039	GCTCTAGATTCCATGGCGGCATCTTCTAATG	
5.	RG042	CTGGTGTTACTACTTCTTTACG	For screening the integration of *TUB2*-GFP cassette at the native locus
6.	PB17	CTCCAGTGAAAAGTTCTTCTC	

Journal of Cell Science • Supplementary information

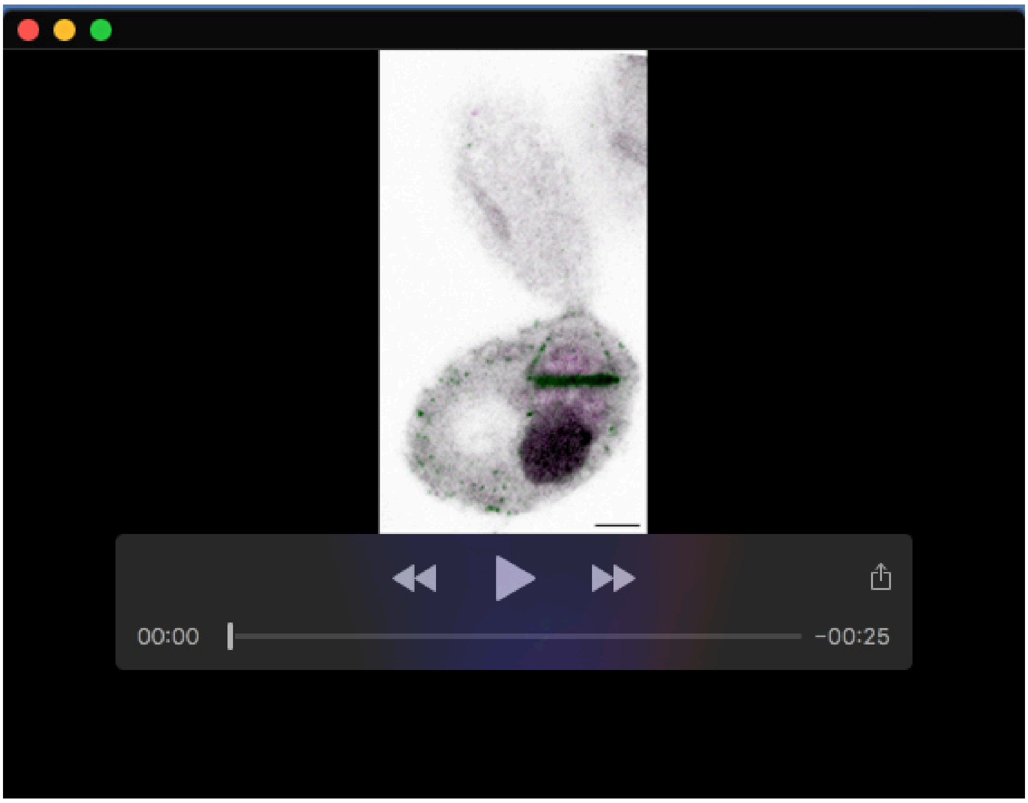

Movie 1. Long aMTs and free cMTs in *C. albicans*. Supplementary

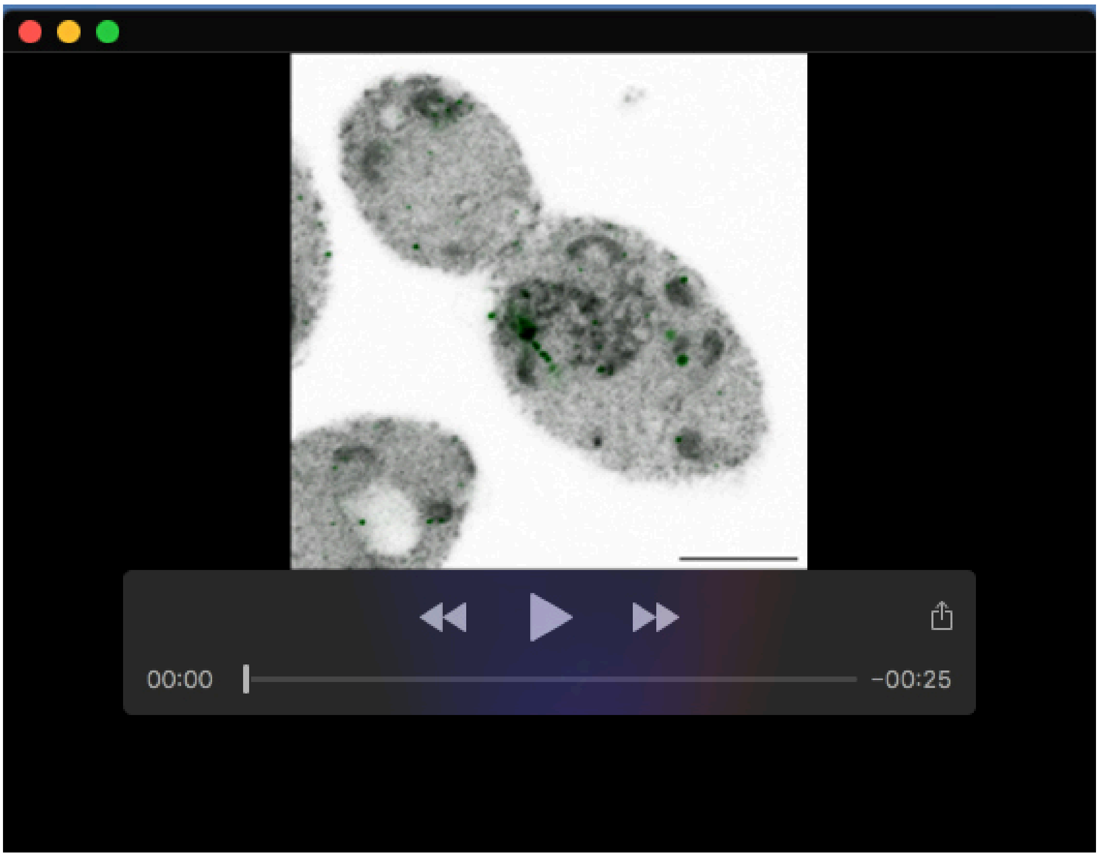

Movie 2. Long aMTs at the pre-anaphase stage in *C. albicans*. Supplementary

Journal of Cell Science • Supplementary information

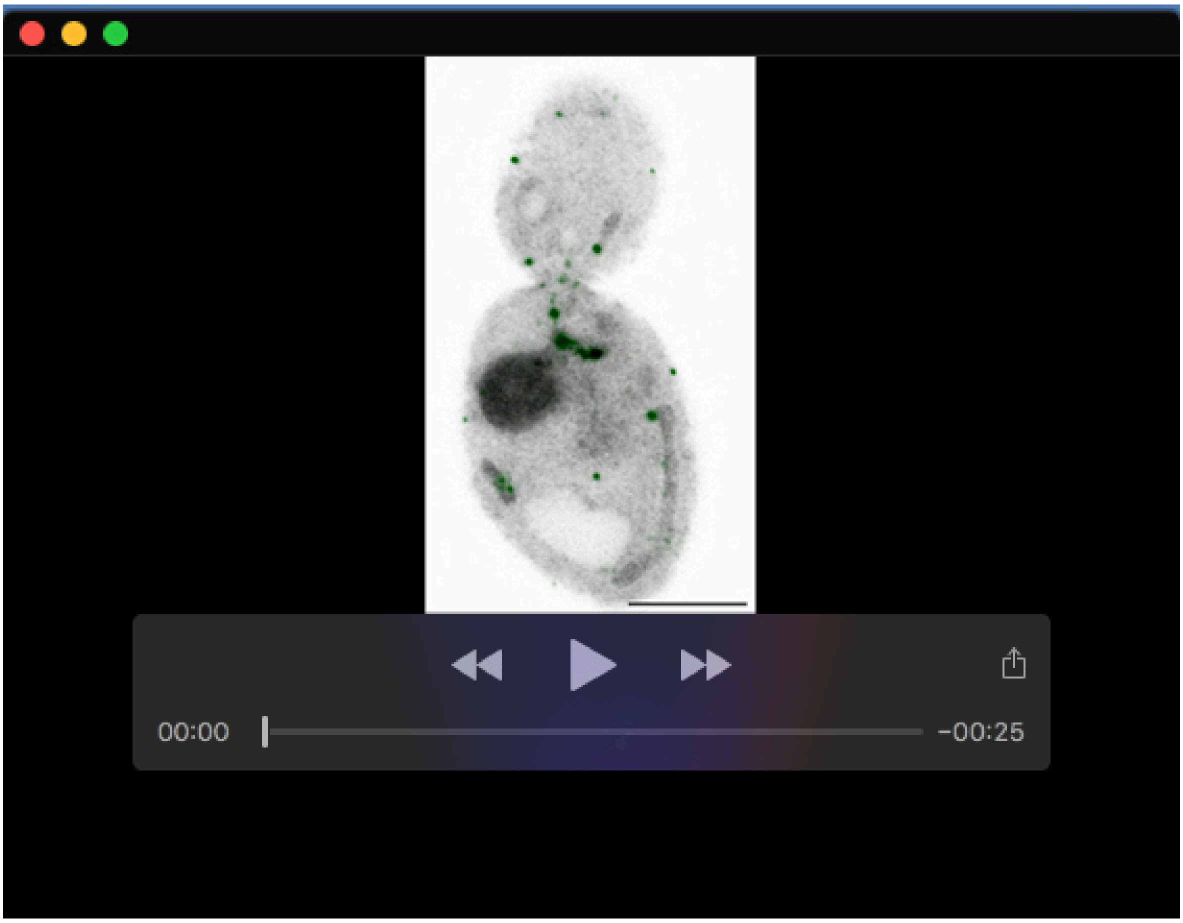

Movie 3. Multiple aMTs at the pre-anaphase stage in *C. albicans*.

Journal of Cell Science • Supplementary information
