# Supplementary material for: Expansion microscopy reveals characteristic ultrastructural features of pathogenic budding yeast species
Source: J Cell Sci. 2024 Sep 9;137(20):jcs262046. doi: 10.1242/jcs.262046 (PMC11423813; doi:10.1242/jcs.262046)
Supplement: Supplementary file 1 [file joces-137-262046_review_history.pdf]

Journal of Cell Science

Expansion microscopy reveals characteristic ultrastructural features of pathogenic budding yeast species

Md Hashim Reza, Srijana Dutta, Rohit Goyal, Hiral Shah, Gautam Dey and Kaustuv Sanyal
DOI: 10.1242/jcs.262046

Editor: Guillaume Jacquemet

Review timeline

Original submission:	21 February 2024
Editorial decision:	2 April 2024
First revision received:	28 May 2024
Editorial decision:	5 July 2024
Second revision received:	9 July 2024
Accepted:	12 July 2024

Original submission

First decision letter

MS ID#: JOCES/2024/262046

MS TITLE: Expansion microscopy reveals unique ultrastructural features of pathogenic budding yeast species

AUTHORS: Md Hashim Reza, Srijana Dutta, Rohit Goyal, Hiral Shah, Gautam Dey, and Kaustuv Sanyal
ARTICLE TYPE: Tools and Resources

We have now reached a decision on the above manuscript.

To see the reviewers' reports and a copy of this decision letter, please go to: https://submit-jcs.biologists.org and click on the 'Manuscripts with Decisions' queue in the Author Area. (Corresponding author only has access to reviews.)

As you will see, the reviewers have mixed opinions about the manuscript and raise a number of substantial criticisms that prevent me from accepting the paper at this stage. If you think that you can deal satisfactorily with the criticisms on revision, I would be pleased to see a revised manuscript. We would then return it to the reviewers.

Please ensure that you clearly highlight all changes made in the revised manuscript. Please avoid using 'Tracked changes' in Word files as these are lost in PDF conversion.

I should be grateful if you would also provide a point-by-point response detailing how you have dealt with the points raised by the reviewers in the 'Response to Reviewers' box. Please attend to all of the reviewers' comments. If you do not agree with any of their criticisms or suggestions please explain clearly why this is so.

Reviewer 1

Advance summary and potential significance to field

© 2024. Published by The Company of Biologists under the terms of the Creative Commons Attribution License (https://creativecommons.org/licenses/by/4.0/).

In this work, Reza and colleagues delivered an interesting article applying expansion microscopy to several fungi species coupled with beautiful imaging. In brief, this study shows the characterisation of organelle organisation in C. albicans and other medically relevant species using expansion microscopy. To achieve this the authors made modifications to the highly used expansion microscopy approach, U-ExM, and applied it to a diverse range of fungal organisms.

Comments for the author

While the overall work is interesting and useful, I have concerns about some of the conclusions provided.
Major concerns arise from the characterisation of the results without enough evidence to support these claims. For this reason, the manuscript requires revisions and I would ask the authors to clarify specific points:

Major comments:

How was the expansion microscopy isotropy assessed? It seems cell size population was compared. Given that comparison and measurements of organelle shape and location inside the cells are stated, the expansion isotropy needs to be assessed by comparing the sample before and after expansion. This is done as a control to guarantee that no deformations are occurring to the sample due to the expansion process. This is particularly important as additional digestion steps are done to the sample. While this represents a considerable amount of work, comparing a few examples as a control should be enough to dispel these concerns.

Throughout the manuscript, several genetically engineered strains are presented (Nop1, Tub2, Tub4, etc) and are not fully validated by other means besides the comparison between their resulting signal and pan-labelling. If this information is available, it is a matter of making it more visible since even after reviewing the supplementary table S1, the information is not clear. This step is critical to guarantee the fidelity of the genetic tagging and the resulting signal.

The use of BodipyTR Ceramide to differentially identify mitochondria is not clear and not fully explained.
When was this staining step done? is this done after fixation and digestion? according to the original publication cited by the authors, this lipid probe is used while the sample is still alive to allow the conversion of the added ceramide into the lipid-rich membranes of the organelles. I would encourage the authors to write a more detailed procedure on the use of this probe as it will be of interest to more researchers.

The statement of a close association between mitochondria and the nucleus (LINE 182) is based on observations without providing quantifiable confirmations. It is expected to find these two organelles close together given their respective size and the reduced available cellular space. If this association was not quantified, the statement should be rephrased.

Minor comments:

LINE 39: “nucleolar and mitochondrial dynamics”.

While we can infer information from fixed samples, unravelling dynamics is often not fully possible without live imaging. The authors are recommended to rephrase this statement to indicate this is done via snapshots of events.

LINE 53: “Since the first documented use of lenses to discover microbial life forms (Porter, 1976)”.

There are earlier documented uses of lenses to observe microbial life forms.

LINE 193: “We also noticed a darkly stained region within the nucleolus of unknown identity (Fig. 3B).”

The arrows in this panel do not seem to be pointing at a specific darker spot in comparison to the rest of the nucleus so it is confusing.

© 2024. Published by The Company of Biologists under the terms of the Creative Commons Attribution License (https://creativecommons.org/licenses/by/4.0/).

LINE 272: “This differential staining was also observed upon NHS-ester labelling, going on to show that similar to S. cerevisiae (Liakopoulos et al., 2003), the old SPB in C. albicans has a higher protein density.”

This statement is unclear. How was the protein density quantified?

LINE 285: “One of the applications of isotropic expansion is the decrowding of the intracellular space which provides the tool to study the effective spatial resolution of proteins.”

Could the authors add a reference for this statement?

LINE 312: “The U-ExM images with tubulin staining also suggested very long aMTs positioned all along the cell cortex (Supplementary Movie 1).”

Is this observation based on multiple examples? if so, the authors should consider providing quantifications.

LINE 384: “We establish U-ExM as a powerful tool to study the cell biology of model and non-model fungal systems.”

The authors should consider toning down this statement as U-ExM has been previously established in fungal systems.

Figures:
Two panels present imaging artefacts: Fig1D late anaphase and Fig2B. While this does not affect the overall story, I would encourage the authors to provide better pictures to maintain the consistently beautiful images you presented in the other panels.

Reviewer 2

Advance summary and potential significance to field

Reza et al, Expansion microscopy reveals unique ultrastructural features of pathogenic budding yeast species.

The authors used expansion microscopy, as proof-of-method, to visualize at 4X expanded resolution of subcellular structures of pathogenic yeasts. While the imaging data are clear, this reviewer found the authors over-interpreted and over-stated the results.

The title and abstract stated “unique ultrastructural features”, “comprehensive map of nucleolar and mitochondrial dynamics through the C. albicans cell cycle”, and “unique side-by-side arrangement of SPBs with a short mitotic spindle and longer astral MTs”.

Expansion microscopy requires fixed and processed cells, therefore words such as “dynamics” of organelles cannot be used to describe the data. The different configurations of nuclei and mitochondria in different cell cycle stages seen in the data can be used to infer dynamics, but the data itself does not show dynamics.

Similarly, as these are fixed cells, it would not be possible to know precisely the period described as pre-anaphase. Thus, C. albicans having unique side-by-side SPBs and short mitotic spindles could represent an earlier time point within pre-anaphase. Indeed, Fig. S1B suggests that the distance between SPBs of C. albicans and S. cerevisiae are similar, with two prominent clusters.

Finally, expansion microscopy as a novel method for S. cerevisiae and S. pombe has previously been published in the JCS. The current paper describes rather incremental progress in applying the same method to pathogenic yeasts.

For these reasons, I cannot recommend publication in the JCS.

© 2024. Published by The Company of Biologists under the terms of the Creative Commons Attribution License (https://creativecommons.org/licenses/by/4.0/).

Comments for the author

-

First revision

Author response to reviewers' comments

Point-by-point responses to reviewers' queries

Reza et al. (2024) MS# JOCES/2024/262046

Reviewer 1

Advance Summary and Potential Significance to Field:

In this work, Reza and colleagues delivered an interesting article applying expansion microscopy to several fungi species coupled with beautiful imaging. In brief, this study shows the characterisation of organelle organisation in *C. albicans* and other medically relevant species using expansion microscopy. To achieve this, the authors made modifications to the highly used expansion microscopy approach, U-ExM, and applied it to a diverse range of fungal organisms.

Comments for the Author:
While the overall work is interesting and useful, I have concerns about some of the conclusions provided. Major concerns arise from the characterisation of the results without enough evidence to support these claims. For this reason, the manuscript requires revisions and I would ask the authors to clarify specific points:
Au: We thank Reviewer 1 for the comprehensive review, encouragement and for considering our work interesting. We also thank the reviewer for appreciating the image quality.

Major comments:
R1.1: How was the expansion microscopy isotropy assessed? It seems cell size population was compared. Given that comparison and measurements of organelle shape and location inside the cells are stated, the expansion isotropy needs to be assessed by comparing the sample before and after expansion. This is done as a control to guarantee that no deformations are occurring to the sample due to the expansion process. This is particularly important as additional digestion steps are done to the sample. While this represents a considerable amount of work, comparing a few examples as a control should be enough to dispel these concerns.
Au: This is a good suggestion. To address this concern, we imaged the same cell, labelled with NHS-ester, before and after expansion, and included the comparison in the revised manuscript as Supporting Figure S1. The results have been included in the revised manuscript. (page 6, lines 161-166).

© 2024. Published by The Company of Biologists under the terms of the Creative Commons Attribution License (https://creativecommons.org/licenses/by/4.0/).

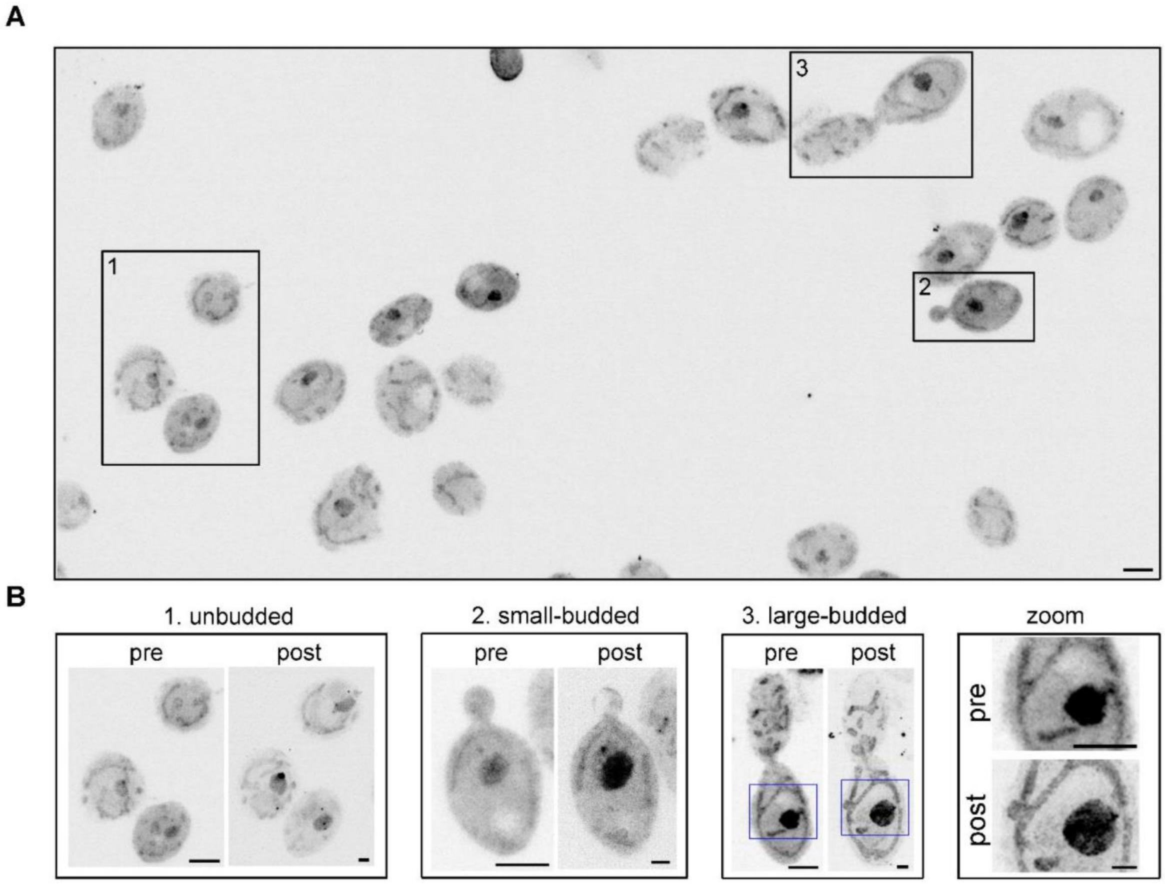

Figure R1. Assessment of isotropic expansion of *Candida albicans* cells before and after expansion. (A) Pre-expansion: Field view of cell wall digested *C. albicans* cells embedded in gel and stained with NHS-ester (grey). Cells compared post-expansion are labelled 1,2, and 3. Scale bar 5 µm. (B) The same *C. albicans* cells were imaged pre- and post-expansion using the same microscope and objective. Images represent cells at various stages of the cell cycle. Blue insets, magnified regions showing the mitochondrial structure at pre- and post-expansion stages. Scale bar 5 µm.

R1.2: Throughout the manuscript, several genetically engineered strains are presented (Nop1, Tub2, Tub4, etc) and are not fully validated by other means besides the comparison between their resulting signal and pan-labelling. If this information is available, it is a matter of making it more visible since even after reviewing the supplementary table S1, the information is not clear. This step is critical to guarantee the fidelity of the genetic tagging and the resulting signal.

Au: Many tagged strains used in this study have already been published (Jaitly et al., 2022, Sankaranarayanan et al., 2023). Specifically, the Tub4-mCherry construct has been previously used to mark the SPBs in *C. albicans*. For the Tub2-GFP Tub4-mCherry tagged *C. albicans*, we have now confirmed the integration of Tub2-GFP at the native locus by PCR and looked at the growth phenotype of the tagged strains with the parent control. This is included as a part of the Supporting Figure S5 (Fig. S5A-C) and is included in the revised manuscript. (page 9, lines 279-281, and page 13, lines 394-402)

© 2024. Published by The Company of Biologists under the terms of the Creative Commons Attribution License (https://creativecommons.org/licenses/by/4.0/).

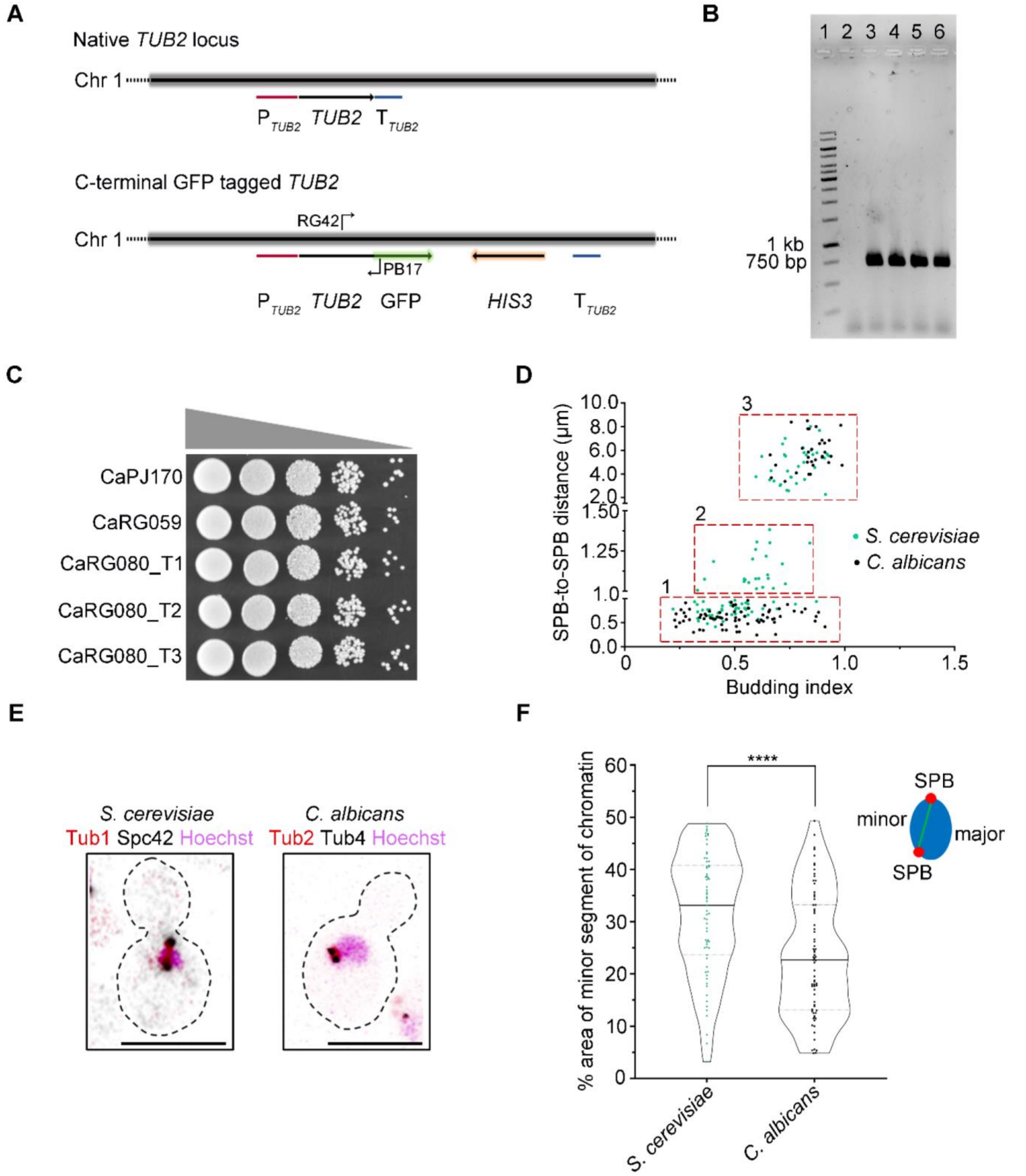

Figure R2. SPB positioning during cell division in *C. albicans*. (A) A schematic representation showing *TUB2* tagging with GFP at the C-terminus at the native locus. Primer pairs RG42/PB17 (~690 bp) were used to confirm the integration of the tagging cassette at the native locus. (B) A 1% agarose gel displaying the screening of the transformants by PCR using primer pairs RG42/PB17. Lane 1: 1 kb DNA ladder, lane 2: parent untagged strain (CaPJ170), lane 3: parent tagged with Tub2-GFP (CaRG059), lane 4-6: parent tagged with Tub2-GFP Tub4-mCherry (CaRG080_T1-T3). (C) Overnight grown *C. albicans* cells (CaPJ170, CaRG059, and CaRG080_T1-T3) were 10-fold serially diluted, and spotted on YPD+uridine. Plates were photographed after incubation at 30°C for 30 h. (D) Scatter plot displaying SPB- to-SPB distance with respect to the budding index in *C. albicans* (black) and *S. cerevisiae* (green). SPB-to-SPB distance above 2 µm represents an anaphase spindle. The red boxes represent three prominent clusters obtained in *S. cerevisiae*. *n*> 100 cells. (E) Maximum intensity projection of *S. cerevisiae*, tagged with GFP-Tub1 and Spc42-mCherry, and *C. albicans* tagged with Tub2-GFP and Tub4-mCherry and co-stained with Hoechst during the pre-anaphase stage. Scale bar 5 µm. (F) Cartoon showing the mitotic spindle (green) between the two

© 2024. Published by The Company of Biologists under the terms of the Creative Commons Attribution License (https://creativecommons.org/licenses/by/4.0/).

SPBs (red) dividing the Hoechst-stained chromatin (blue) area into two unequal segments (minor and major) as visually observed in a 2D-projected image. Violin plot showing percent area covered by the minor segment of Hoechst-stained chromatin in *C. albicans* and *S. cerevisiae*. *n*> 70 cells. Statistical analysis was done by Unpaired *t*-test with Welch's correction (****p<0.0001).

In terms of Tub2-GFP, the localisation signals are in line with the previously published literature (Finley and Berman, 2005).
The imaging of the nucleolus was carried out using an anti-Nop1 antibody (Anti-Fibrillarin 442 antibody [38F3]) - Nucleolar Marker (ab4566) and hence, no genetic manipulation was performed to obtain the signals. We have included the details of each strain used in this study in Supplementary Table S1.

R1.3: The use of BodipyTR Ceramide to differentially identify mitochondria is not clear and not fully explained. When was this staining step done? is this done after fixation and digestion? According to the original publication cited by the authors, this lipid probe is used while the sample is still alive to allow the conversion of the added ceramide into the lipid-rich membranes of the organelles. I would encourage the authors to write a more detailed procedure on the use of this probe as it will be of interest to more researchers.

Au: The staining with Bodipy^{TR} was carried out after fixation and digestion. We have applied the protocol to several species (Shah et al., 2024). We have included a more detailed procedure for Bodipy^{TR} staining in the materials and methods section. (page 15, lines 481-485)

R1.4: The statement of a close association between mitochondria and the nucleus (LINE 182) is based on observations without providing quantifiable confirmations. It is expected to find these two organelles close together given their respective size and the reduced available cellular space. If this association was not quantified, the statement should be rephrased.
Au: We removed the comment from the revised manuscript because it was an incidental observation. Since it does not impact our overall claim and story, we are not keeping the statement of the close association between mitochondria and the nucleus. We thank the reviewer for the suggestion.

Minor comments:
R1.5: LINE 39: "nucleolar and mitochondrial dynamics". While we can infer information from fixed samples, unravelling dynamics is often not fully possible without live imaging. The authors are recommended to rephrase this statement to indicate this is done via snapshots of events.
Au: As suggested by the reviewer, we have rephrased the statement to "NHS-ester pan- labelling in combination with immunofluorescence (IF) *via* snapshots of various mitotic stages provided a comprehensive map of nucleolar and mitochondrial segregation dynamics and enabled the resolution of inner and outer plaque of spindle pole bodies (SPBs).". (page 3, lines 66-69)

R1.6: LINE 53: "Since the first documented use of lenses to discover microbial life forms (Porter, 1976)". There are earlier documented uses of lenses to observe microbial life forms.
Au: We have modified this statement to "Since the first documented use of lenses to discover microbial life forms by Hooke and Leeuwenhoek during 1665-1683 (Gest, 2004), various advancements have been brought about to improve the resolution of imaging". (page 4, lines 93-95)

R1.7: LINE 193: "We also noticed a darkly stained region within the nucleolus of unknown identity (Fig. 3B)." The arrows in this panel do not seem to be pointing at a specific darker spot in comparison to the rest of the nucleus so it is confusing.
Au: We thank the reviewer for pointing this out. We have now included a revised figure which marks the darkly stained region and is included as Supporting Figure S2 in the revised manuscript. (page 7, lines 202-203)

© 2024. Published by The Company of Biologists under the terms of the Creative Commons Attribution License (https://creativecommons.org/licenses/by/4.0/).

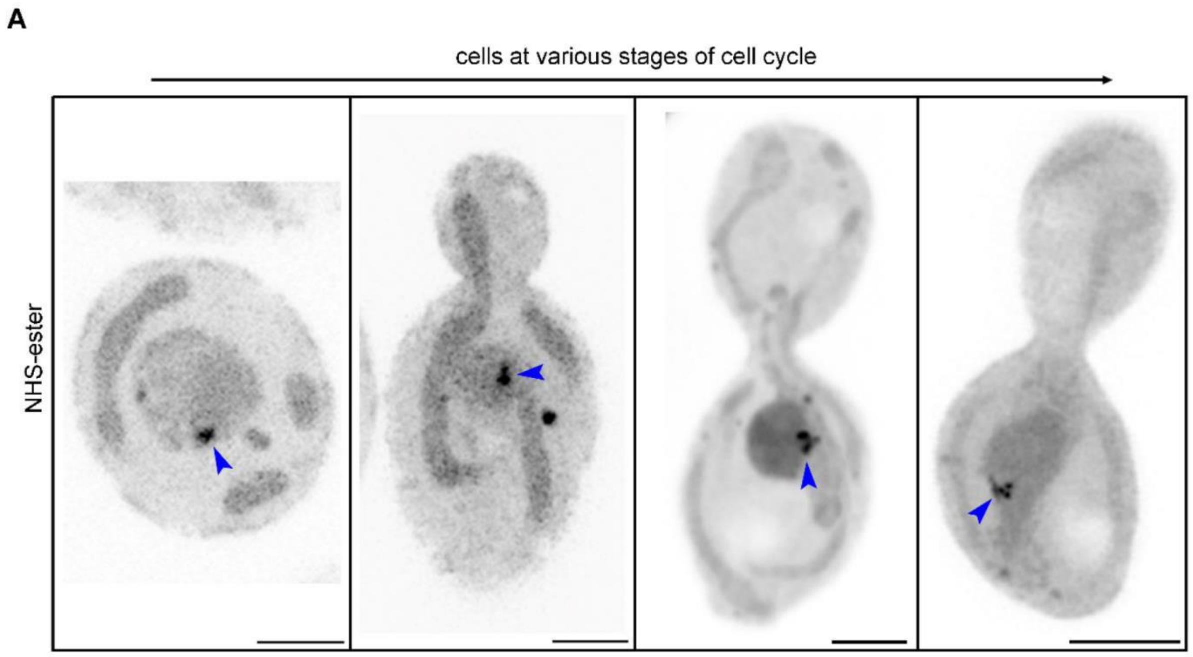

Figure R3. U-ExM reveals a darkly stained region within the nucleolus. (A) Maximum intensity projection of *C. albicans* cells stained with NHS-ester showing the darkly stained region (blue arrowheads) within the nucleolus. Scale bar 5 µm.

R1.8: LINE 272: “This differential staining was also observed upon NHS-ester labelling, going on to show that similar to *S. cerevisiae* (Liakopoulos et al., 2003), the old SPB in *C. albicans* has a higher protein density.” This statement is unclear. How was the protein density quantified?

Au: We have now included the quantification to differentiate between the two SPBs by probing for Spc110-GFP and NHS-ester signal intensity and reported it as a part of Figure 4 (Fig. 4A- C) in the revised manuscript. Steps for quantification are described in methods. (page 8, lines 237-244)

© 2024. Published by The Company of Biologists under the terms of the Creative Commons Attribution License (https://creativecommons.org/licenses/by/4.0/).

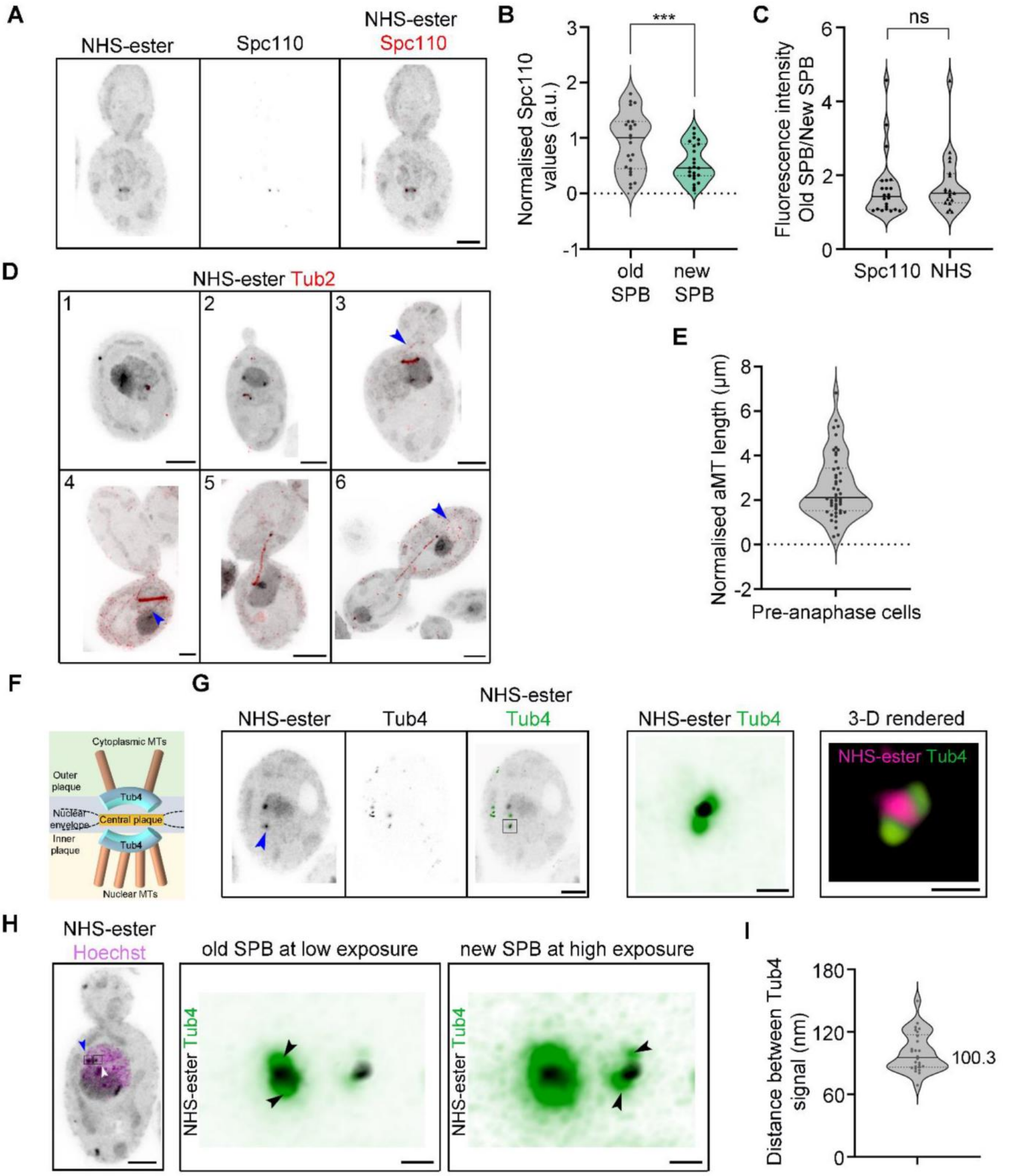

Figure R4. SPB organisation in *C. albicans*. (A) Maximum intensity projection of *C. albicans* cells, co-stained with NHS-ester (grey) and anti-GFP (Spc110-GFP, red). Scale bar 5 µm. (B) The violin plot displays Spc110 signal intensities. The signal was quantified and normalised to the mean of SPB1 (old SPB). *n*=23 expanded cells analysed. Statistical analysis was done using a paired *t*-test (***p=0.0005). (C) The violin plot displays the ratio of fluorescent intensities between old and new SPB as determined by NHS-ester signal intensities. The signal was quantified and the old SPB signal was normalised to the signal from the new SPB. *n*=22 (Spc110) and 19 (NHS-ester) expanded cells analysed. Statistical analysis was done using an Unpaired *t*-test. (D) Maximum intensity projection of *C. albicans* cells co-stained with NHS- ester (grey) and anti-GFP (Tub2-GFP, red), at various stages of the cell cycle (1, 2, 3, 4, 5, and 6). The blue arrowheads mark the astral microtubules (aMTs). Scale bar 5 µm. (E) A violin plot showing the distribution of aMT length at the pre-anaphase stage of the cell cycle in expanded *C. albicans* cells. *n*=47 aMTs analysed. (F) Schematic showing the spatial position of Tub4 at the SPB as reported from *S. cerevisiae*. (G) A representative maximum-intensity projection of *C. albicans* cells co-stained with NHS-ester (grey) and anti-GFP

© 2024. Published by The Company of Biologists under the terms of the Creative Commons Attribution License (https://creativecommons.org/licenses/by/4.0/).

(Tub4-GFP, green) in unbudded cells. Scale bar 5 µm. Inset, a magnified region depicted by a black- bordered square and a 3-D rendered image of the magnified region is also shown. Scale bar 125 nm. (H) Maximum-intensity projection of *C. albicans* cells co-stained with NHS-ester (grey) and Hoechst (magenta) at the pre-anaphase stage of the cell cycle. Scale bar 5 µm. The blue and white arrowhead represents the old and new SPBs, based on signal intensity. Inset, a magnified region is shown for the two SPBs. The first inset highlights the Tub4 arrangement (black arrowheads) at the old SPB, visible at low-intensity exposure. The second inset highlights the Tub4 arrangement (black arrowheads) at the new SPB visible only at high- intensity exposure. Scale bar 125 nm. (I) A violin plot displaying the distance between two Tub4 fluorescent signals rescaled after expansion (N=2, *n*=26).

R1.9: LINE 285: "One of the applications of isotropic expansion is the decrowding of the intracellular space which provides the tool to study the effective spatial resolution of proteins." Could the authors add a reference for this statement?
Au: We have included the reference for this statement. (page 9, lines 261-264)

R1.10: LINE 312: "The U-ExM images with tubulin staining also suggested very long aMTs positioned all along the cell cortex (Supplementary Movie 1)." Is this observation based on multiple examples? if so, the authors should consider providing quantifications.
Au: We have now quantified the length of the aMTs from multiple cells at the pre-anaphase stage which is now included as a part of Figure 4 (Fig. 4E) in the revised manuscript. (page 8, lines 250-257)

© 2024. Published by The Company of Biologists under the terms of the Creative Commons Attribution License (https://creativecommons.org/licenses/by/4.0/).

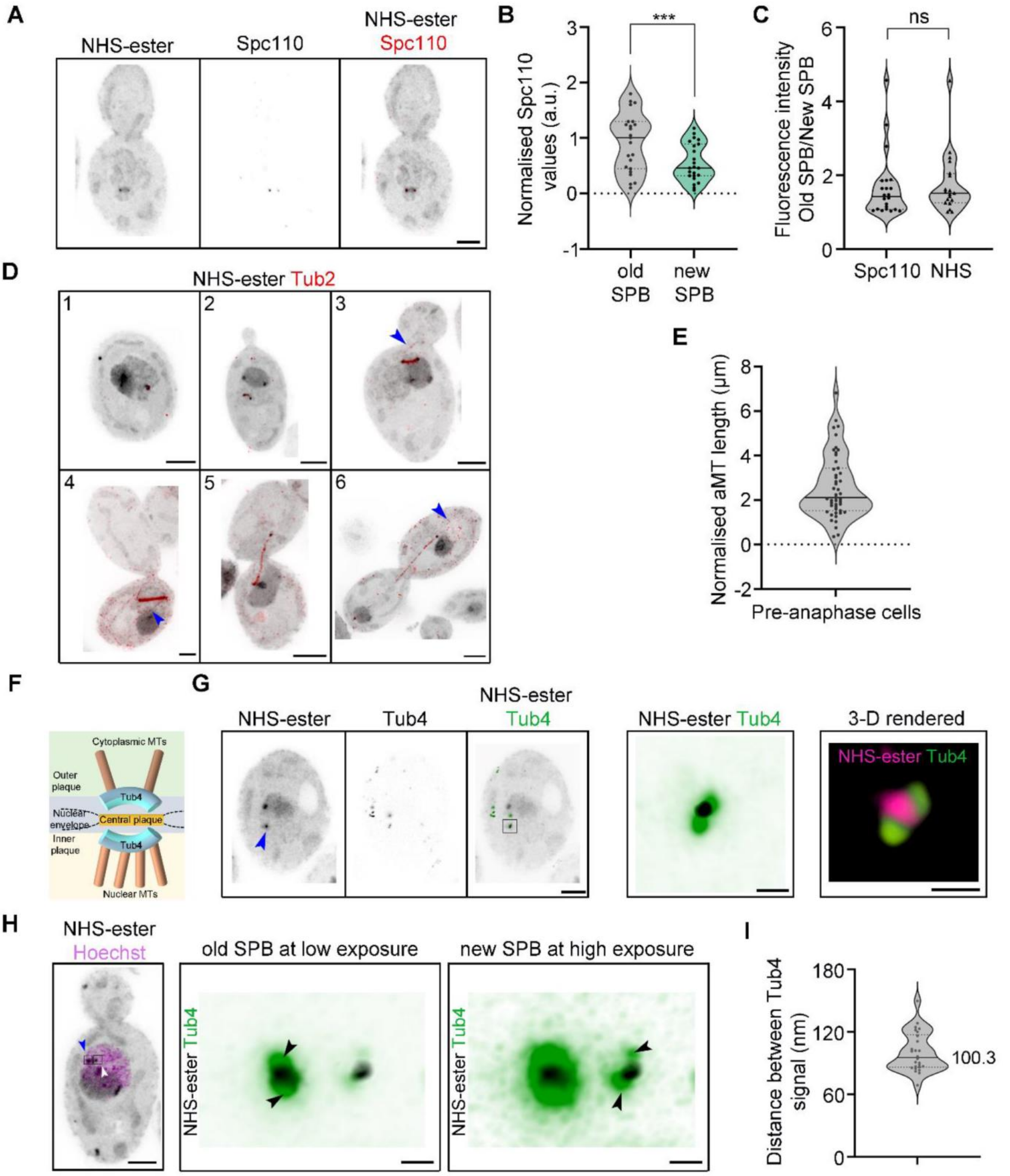

Figure R5. SPB organisation in *C. albicans*. (A) Maximum intensity projection of *C. albicans* cells, co-stained with NHS-ester (grey) and anti-GFP (Spc110-GFP, red). Scale bar 5 µm. (B) The violin plot displays Spc110 signal intensities. The signal was quantified and normalised to the mean of SPB1 (old SPB). *n*=23 expanded cells analysed. Statistical analysis was done using a paired *t*-test (***p=0.0005). (C) The violin plot displays the ratio of fluorescent intensities between old and new SPB as determined by Spc110 and NHS-ester signal intensities. The signal was quantified and the old SPB signal was normalised to the signal from the new SPB. *n*=22 (Spc110) and 19 (NHS-ester) expanded cells analysed. Statistical analysis was done using Unpaired *t*-test. (D) Maximum intensity projection of *C. albicans* cells co-stained with NHS- ester (grey) and anti-GFP (Tub2-GFP, red), at various stages of the cell cycle (1, 2, 3, 4, 5, and 6). The blue arrowheads mark the astral microtubules (aMTs). Scale bar 5 µm. (E) A violin plot showing the distribution of aMT length at the pre-anaphase stage of the cell cycle in expanded *C. albicans* cells. *n*=47 aMTs analysed. (F) Schematic showing the spatial position of Tub4 at the SPB as reported from *S. cerevisiae*. (G) A representative maximum-intensity projection of *C. albicans* cells co-stained with NHS-ester

© 2024. Published by The Company of Biologists under the terms of the Creative Commons Attribution License (https://creativecommons.org/licenses/by/4.0/).

(grey) and anti-GFP (Tub4-GFP, green) in unbudded cells. Scale bar 5 µm. Inset, a magnified region depicted by a black- bordered square and a 3-D rendered image of the magnified region is also shown. Scale bar 125 nm. (H) Maximum-intensity projection of *C. albicans* cells co-stained with NHS-ester (grey) and Hoechst (magenta) at the pre-anaphase stage of the cell cycle. Scale bar 5 µm. The blue and white arrowhead represents the old and new SPBs, based on signal intensity. Inset, a magnified region is shown for the two SPBs. The first inset highlights the Tub4 arrangement (black arrowheads) at the old SPB, visible at low-intensity exposure. The second inset highlights the Tub4 arrangement (black arrowheads) at the new SPB visible only at high- intensity exposure. Scale bar 125 nm. (I) A violin plot displaying the distance between two Tub4 fluorescent signals rescaled after expansion (N=2, *n*=26).

R1.11: LINE 384: “We establish U-ExM as a powerful tool to study the cell biology of model and non-model fungal systems.” The authors should consider toning down this statement as U- ExM has been previously established in fungal systems.
Au: As suggested by the reviewer, we have modified the statement to “We extended this protocol to expand its application to study the cell biology of some of the non-model medically relevant human fungal pathogens”. (page 11, lines 334-336)

R1.12: Figures: Two panels present imaging artefacts: Fig1D late anaphase and Fig2B. While this does not affect the overall story, I would encourage the authors to provide better pictures to maintain the consistently beautiful images you presented in the other panels.
Au: We thank the reviewer for appreciating the figure quality and pointing out artefacts for the late anaphase cell in Figure 1D and Figure 2B. As suggested, we have processed the image again for the late anaphase cell in Figure 1D.

© 2024. Published by The Company of Biologists under the terms of the Creative Commons Attribution License (https://creativecommons.org/licenses/by/4.0/).

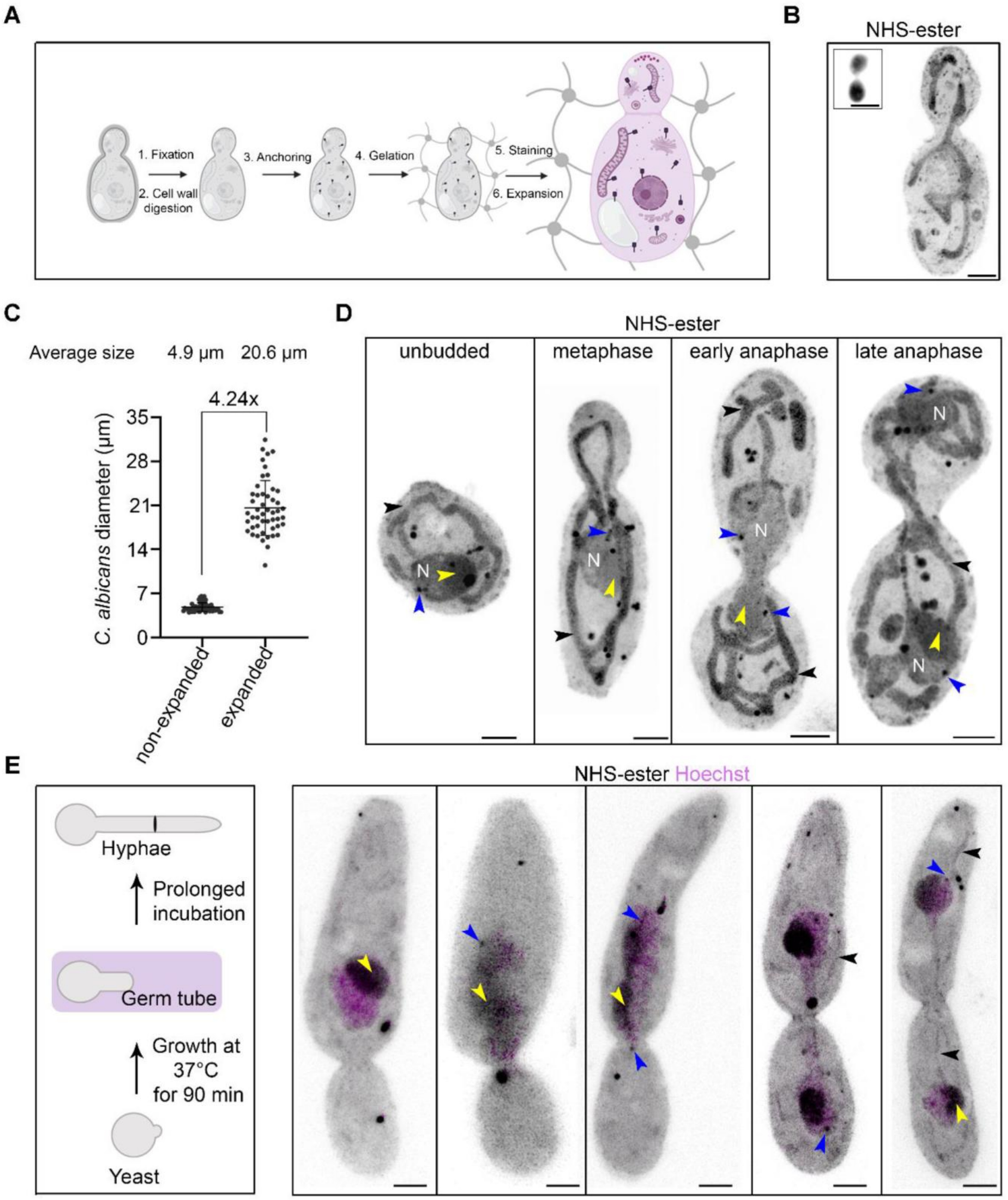

Figure R6. Pan-labelling of proteome displays sub-cellular organisation in expanded *Candida albicans* cells. (A) Schematic displaying different steps of ultrastructure expansion microscopy (U-ExM) protocol in *C. albicans*, which includes fixation (1), cell wall digestion (2), anchoring (3), gelation (4), staining (5), and expansion (6). (B) Representative confocal image of *C. albicans* cells post-expansion, pan-labelled with NHS-ester and displayed as maximum intensity projections. The inset shows a non-expanded cell. Scale bar 5 µm. (C) Scatter plot displaying the 4.24x expansion factor based on measurement of the diameter (longer axis) of unbudded cells of *C. albicans* before (*n*=54) and after (*n*=49) expansion. Error bars show mean±s.d. (D) Representative images of *C. albicans* pan-labelled with NHS-ester showing sub-cellular organisation at various stages of the cell cycle. The black and blue arrowheads represent mitochondria and SPBs, respectively. The nucleus is marked with N and the nucleolus is marked with a yellow arrowhead. Scale bar 5 µm. (E) Schematic showing morphogenetic changes during the yeast-to-hyphal transition. Cells at the germ tube stage (magenta) were taken forward for U-ExM. Representative images of *C. albicans* pan-labelled with NHS-ester (grey) and co-stained with Hoechst (magenta) showing sub-cellular

© 2024. Published by The Company of Biologists under the terms of the Creative Commons Attribution License (https://creativecommons.org/licenses/by/4.0/).

organisation during cell division after hyphal induction. The black, blue, and yellow arrowheads represent mitochondria, SPBs, and nucleolus respectively. Scale bar 5 µm.

We also reprocessed the images in Figure 2B and replaced the last image for a better representation and without any artefacts.

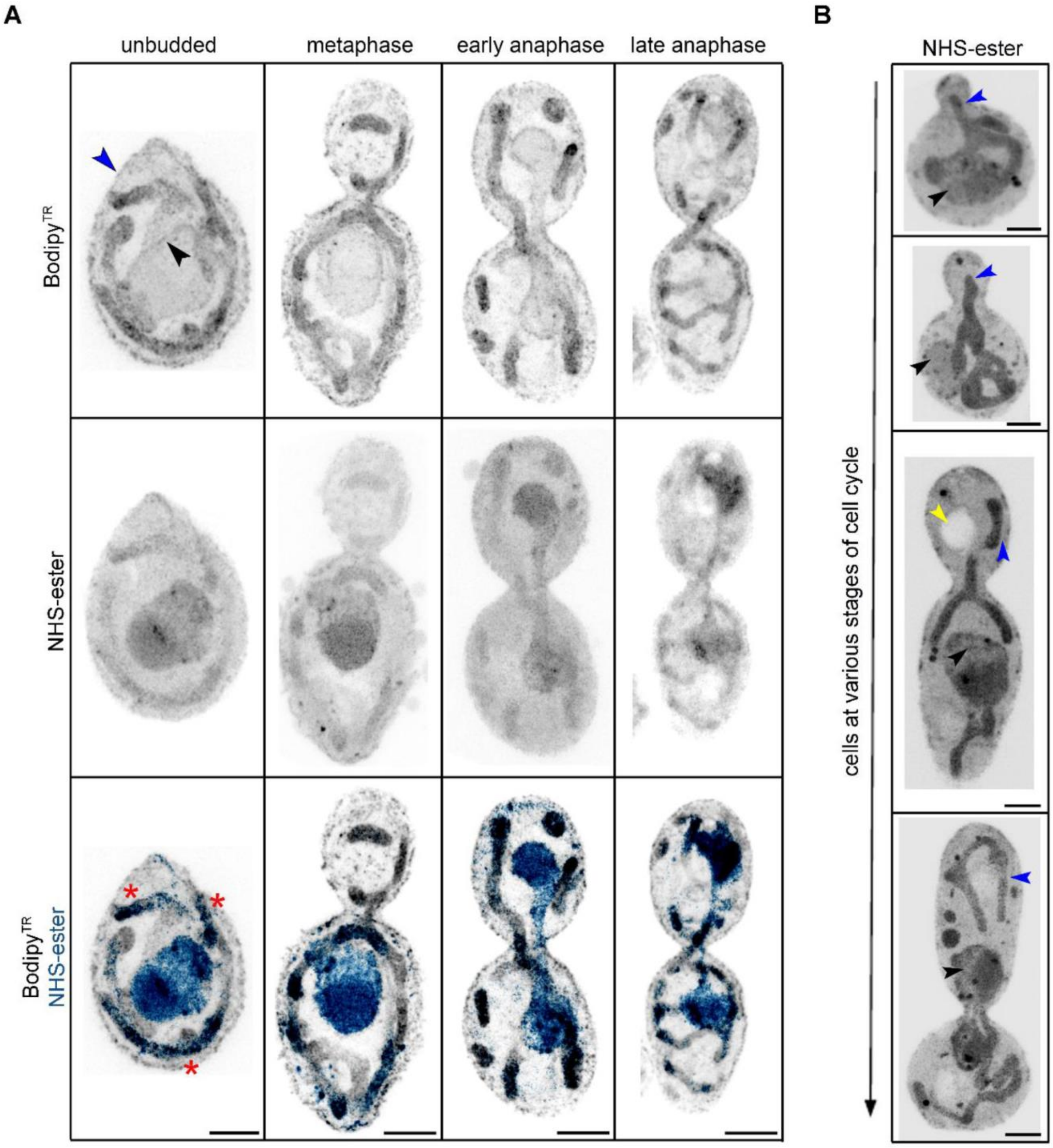

Figure R7. The tubular mitochondrial network segregates into daughter cells before nuclear segregation in *C. albicans*. (A) Maximum intensity projection of *C. albicans* cells co- stained with Bodipy^{TR} (grey) and NHS-ester (blue), during cell division. The blue and black arrowheads mark the cell and nuclear membrane, respectively. Red asterisks mark the mitochondria labelled with both Bodipy^{TR} and NHS-ester. Scale bar 5 µm. (B) Maximum intensity projection of *C. albicans* cells stained with NHS-ester (grey), during the cell cycle displays segregation of mitochondria (blue arrowheads) before nuclear (black arrowheads) segregation between the two cells. The yellow arrowhead marks the unlabelled vacuole in the cell. Scale bar 5 µm.

To maintain the consistency of the image quality, we went ahead and repeated the immunostaining of Nop1 using anti-Nop1 antibodies and included the image as a part of Figure

© 2024. Published by The Company of Biologists under the terms of the Creative Commons Attribution License (https://creativecommons.org/licenses/by/4.0/).

3 (Fig. 3B) in the revised manuscript. (page 7, lines 215-218)

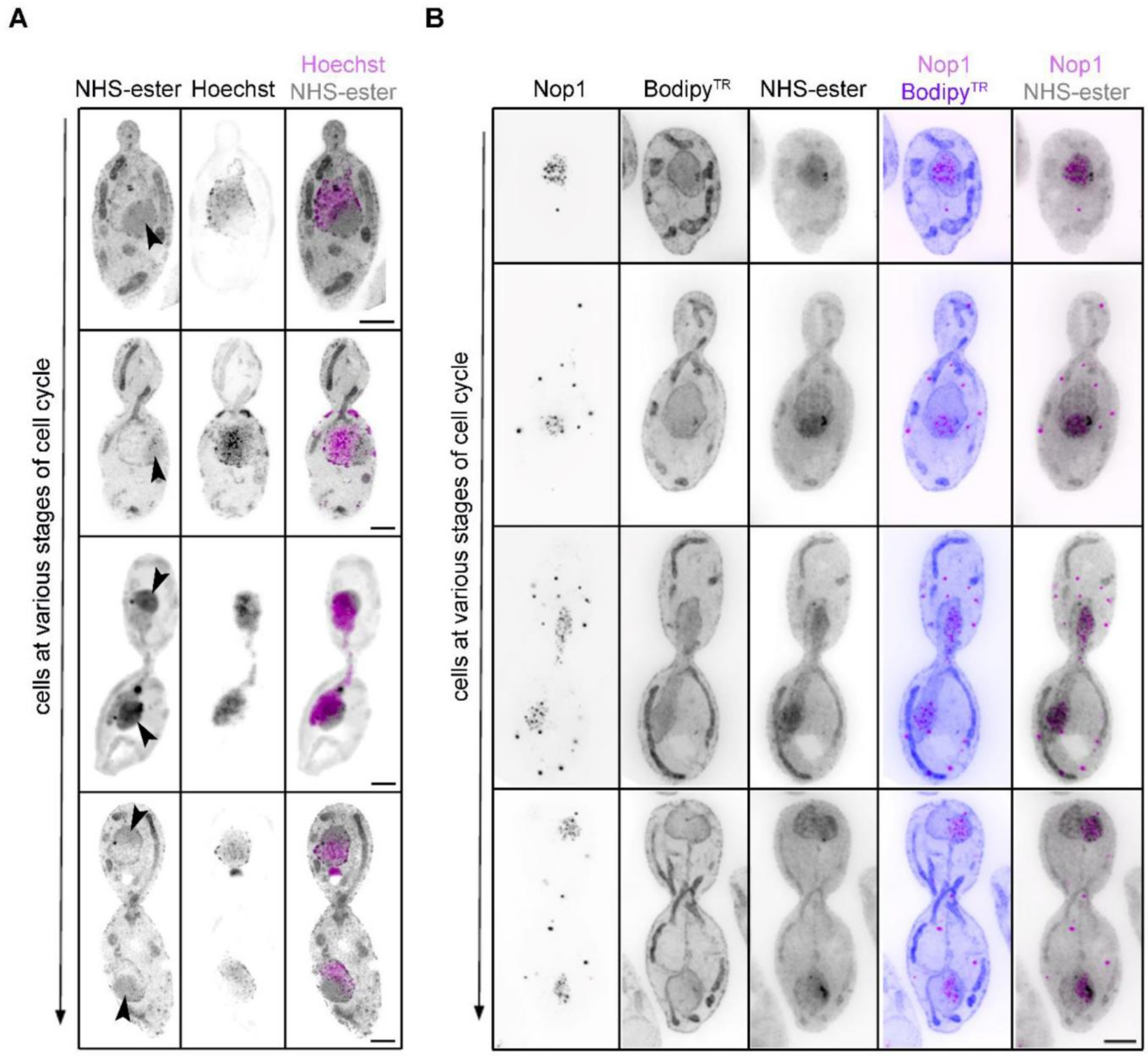

Figure R8. Nucleolar segregation during cell division in *C. albicans*. (A) Maximum intensity projection of *C. albicans* cells co-stained with Hoechst (magenta) and NHS-ester (grey) during cell division. The black arrowheads mark the nucleolus. Scale bar 5 µm. (B) Maximum intensity projection of *C. albicans* cells co-stained with NHS-ester (grey), Bodipy^{TR} (blue) and Nop1 Abs (magenta) at various stages of the cell cycle. Scale bar 5 µm.

Reviewer 2

Advance Summary and Potential Significance to Field:

Reza et al, Expansion microscopy reveals unique ultrastructural features of pathogenic budding yeast species. The authors used expansion microscopy, as proof-of-method, to visualize at 4X expanded resolution of subcellular structures of pathogenic yeasts. While the imaging data are clear, this reviewer found the authors over-interpreted and over-stated the results.

The title and abstract stated “unique ultrastructural features”, “comprehensive map of nucleolar and mitochondrial dynamics through the *C. albicans* cell cycle”, and “unique side-by-side arrangement of SPBs with a short mitotic spindle and longer astral MTs”.

Au: We thank Reviewer 2 for providing constructive input for our work. We have modified the

© 2024. Published by The Company of Biologists under the terms of the Creative Commons Attribution License (https://creativecommons.org/licenses/by/4.0/).

manuscript and addressed all the concerns raised.

R2.1: Expansion microscopy requires fixed and processed cells, therefore words such as "dynamics" of organelles cannot be used to describe the data. The different configurations of nuclei and mitochondria in different cell cycle stages seen in the data can be used to infer dynamics, but the data itself does not show dynamics.
Au: As pointed out by both reviewers, we agree that these images represent the snapshots of various stages of the cell cycle and therefore can be used to infer the dynamics of various organelles. As suggested, we have clearly mentioned this in the revised manuscript, "Based on the budding index and having prior knowledge of cell cycle progression in *C. albicans* (Berman, 2006, Sreekumar et al., 2021), the snapshots captured at various stages of mitosis do enable a fair idea of the organellar dynamics". (page 7, lines 205-207)

R2.2: Similarly, as these are fixed cells, it would not be possible to know precisely the period described as pre-anaphase. Thus, *C. albicans* having unique side-by-side SPBs and short mitotic spindles could represent an earlier time point within pre-anaphase. Indeed, Fig. S1B suggests that the distance between SPBs of *C. albicans* and *S. cerevisiae* are similar, with two prominent clusters.
Au: We thank the reviewer for the suggestion to validate the pre-anaphase spindle lengths in *C. albicans*. We would like to bring to the reviewer's notice that our claim of a unique side-by- side arrangement of SPBs in *C. albicans* was not based on fixed cells. For this analysis, the log-phase grown cells were stained with Hoechst. Additionally, the analysis of the SPB-to-SPB distance, as shown in Fig. S1B (revised Fig. S5D), suggests a subpopulation of *S. cerevisiae* cells which have longer pre-anaphase spindle spanning from 1-1.5 µm (Box 2, marked in red), which are not present in *C. albicans*. This analysis was carried out for more than 100 cells of each organism and thus represents a distribution.
However, to probe further, we have now carried out live-cell movies starting from small- budded cells and followed mitotic spindle dynamics till 3-4 µm spindle both for *C. albicans* and *S. cerevisiae* at 45-sec time intervals. The results from this analysis are now presented in Figure 5 in the revised manuscript. (pages 9-10, lines 291-299)

© 2024. Published by The Company of Biologists under the terms of the Creative Commons Attribution License (https://creativecommons.org/licenses/by/4.0/).

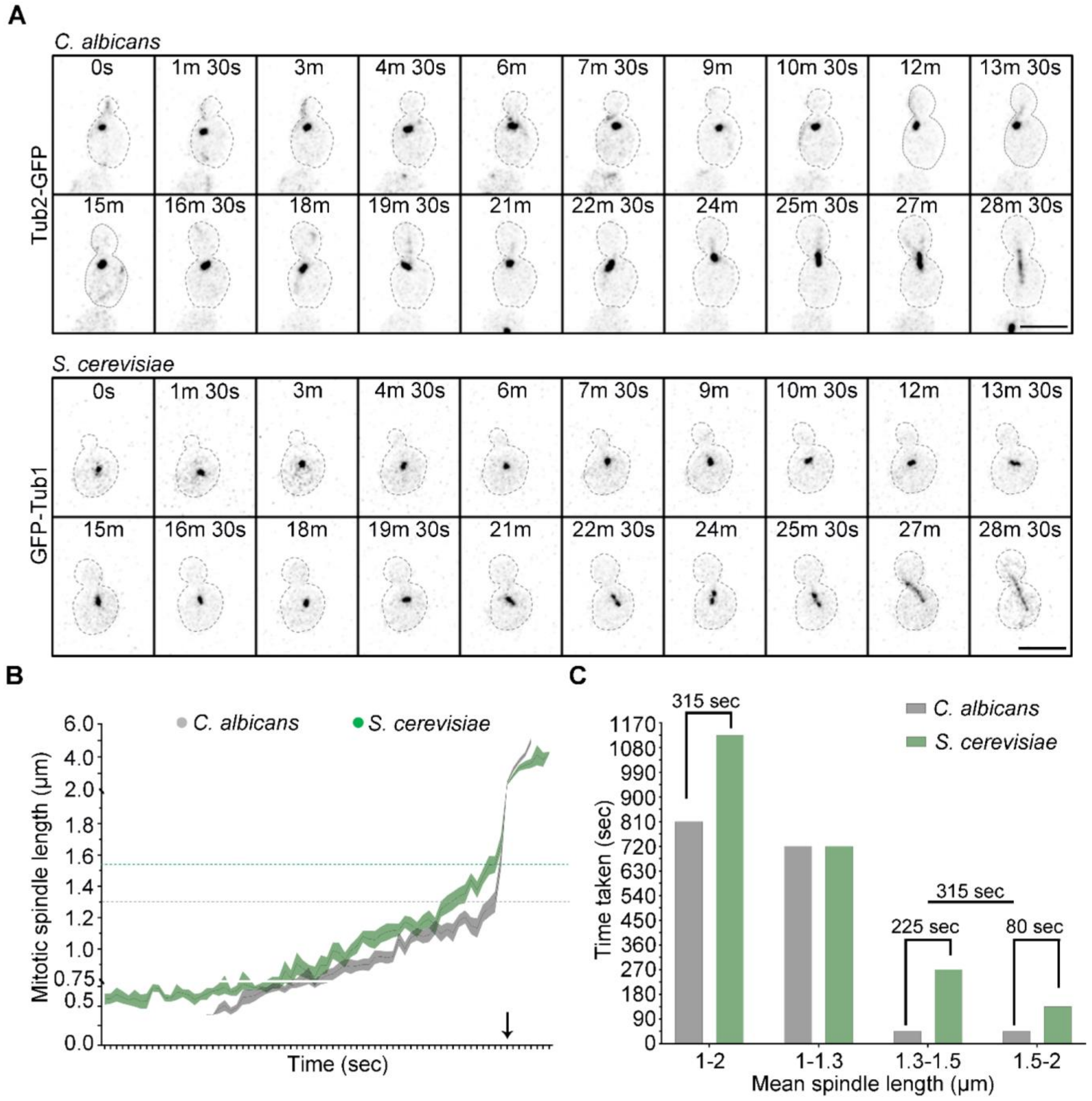

Figure R9. *C. albicans* displays a faster anaphase onset with a shorter pre-anaphase spindle. (A) A montage of time-lapse images showing dynamics of the mitotic spindle, tagged with GFP, during the cell cycle in *C. albicans* (*top*) and *S. cerevisiae* (*bottom*) at 30°C. Scale bar 5 µm. (B) A line graph displaying the mitotic spindle length distribution during cell cycle progression in *C. albicans* (grey) and *S. cerevisiae* (green). Anaphase onset is marked by horizontal lines, grey (*C. albicans*) and green (*S. cerevisiae*). *n*=25 and 23 live-cell movies for *C. albicans* and *S. cerevisiae*, respectively. The first time point corresponding to >=2 µm spindle length (black arrow) was used as a reference point to align all the live-cell movie data. The major ticks on the *x*-axis represent 45-sec intervals. The plot represents mean (black line) ±s.e.m. (C) Bar graph displaying the time taken (*y*-axis) for reaching maximum mean spindle length in *C. albicans* (grey) and *S. cerevisiae* (green) for different categories. The difference in time taken for each category of mean spindle length between *C. albicans* and *S. cerevisiae* is mentioned in the graph.

Our analysis suggests a spindle length of 1.3 and 1.56 µm in *C. albicans* and *S. cerevisiae*, respectively marking anaphase onset (Fig. R9B). We believe that due to the rapid elongation of the mitotic spindle, the cell population between 1.1-2 µm and 1.4-2 µm corresponding to *C. albicans* and *S. cerevisiae*, respectively are difficult to capture in still images (Fig. R10D). Taken together, a shorter pre-anaphase spindle translates to a lower nuclear area coverage and hence side-by-side SPB arrangement in *C. albicans*.

© 2024. Published by The Company of Biologists under the terms of the Creative Commons Attribution License (https://creativecommons.org/licenses/by/4.0/).

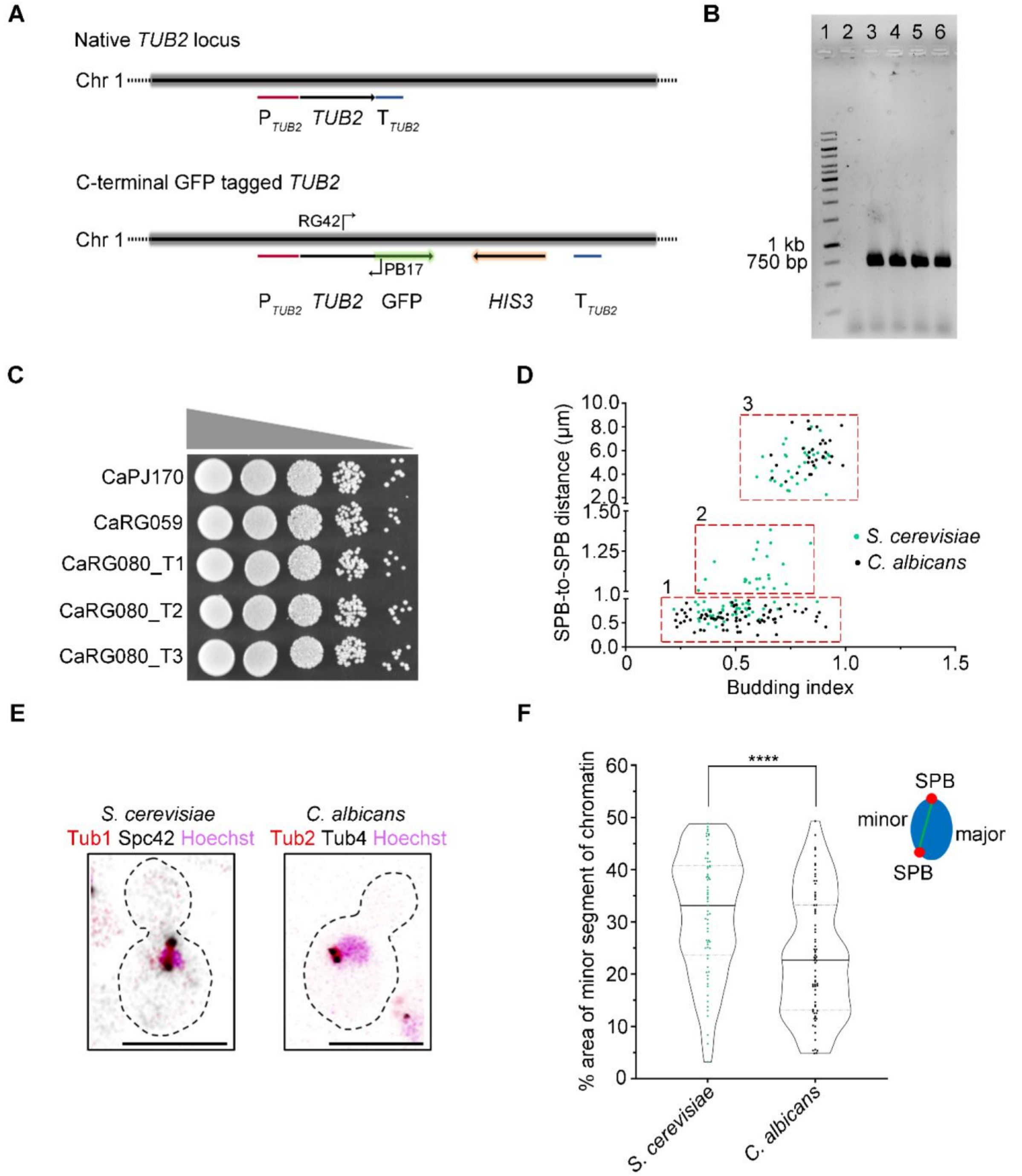

Figure R10. SPB positioning during cell division in *C. albicans*. (A) A schematic representation showing *TUB2* tagging with GFP at the C-terminus at the native locus. Primer pairs RG42/PB17 (~690 bp) were used to confirm the integration of the tagging cassette at the native locus. (B) A 1% agarose gel displaying the screening of the transformants by PCR using primer pairs RG42/PB17. Lane 1: 1 kb DNA ladder, lane 2: parent untagged strain (CaPJ170), lane 3: parent tagged with Tub2-GFP (CaRG059), lane 4-6: parent tagged with Tub2-GFP Tub4-mCherry (CaRG080_T1-T3). (C) Overnight grown *C. albicans* cells (CaPJ170, CaRG059, and CaRG080_T1-T3) were 10-fold serially diluted, and spotted on YPD+uridine. Plates were photographed after incubation at 30°C for 30 h. (D) Scatter plot displaying SPB- to-SPB distance with respect to the budding index in *C. albicans* (black) and *S. cerevisiae* (green). SPB-to-SPB distance above 2 µm represents an anaphase spindle. The red boxes represent three prominent clusters obtained in *S. cerevisiae*. *n*> 100 cells. (E) Maximum intensity projection of *S. cerevisiae*, tagged with GFP-Tub1 and Spc42-mCherry, and *C. albicans* tagged with Tub2-GFP and Tub4-mCherry and co-stained with Hoechst during the pre-anaphase stage. Scale bar 5 µm. (F) Cartoon showing the mitotic spindle (green) between the two

© 2024. Published by The Company of Biologists under the terms of the Creative Commons Attribution License (https://creativecommons.org/licenses/by/4.0/).

SPBs (red) dividing the Hoechst-stained chromatin (blue) area into two unequal segments (minor and major) as visually observed in a 2D-projected image. Violin plot showing percent area covered by the minor segment of Hoechst-stained chromatin in *C. albicans* and *S. cerevisiae*. *n*> 70 cells. Statistical analysis was done by Unpaired *t*-test with Welch's correction (****p<0.0001).

R2.3: Finally, expansion microscopy as a novel method for *S. cerevisiae* and *S. pombe* has previously been published in the JCS. The current paper describes rather incremental progress in applying the same method to pathogenic yeasts. For these reasons, I cannot recommend publication in the JCS.

Au: The fungal kingdom is vastly diverse, with the various yeasts in our study spanning a broad phylogenetic range of 600 – 700 million years. The utilisation of available molecular tools for understanding these human pathogens is greatly challenged by the varied range of cellular architectural differences. Although the initial protocol for *S. cerevisiae* and *S. pombe* (Hinterndorfer et al., 2022) served as a starting point for our investigation, our results demonstrate that the protocol required specific and significant modifications to successfully expand pathogenic yeasts. Further, we also show how this tool of U-ExM can be applied to study SPB configurations across a diverse set of fungal species solely based on pan-labelling of the proteome. This observation provides powerful insights into conserved ultrastructural features of pathogenic yeasts without the need for genetic manipulation to visualise the events.

References

JAITLY, P., LEGRAND, M., DAS, A., PATEL, T., CHAUVEL, M., MAUFRAIS, C., D'ENFERT, C. & SANYAL, K. 2022. A phylogenetically-restricted essential cell cycle progression factor in the human pathogen *Candida albicans*. *Nat Commun,* 13, 4256.

SANKARANARAYANAN, S. R., POLISETTY, S. D., DAS, K., DUMBREPATIL, A., MEDINA-PRITCHARD, B., SINGLETON, M., JEYAPRAKASH, A. A. & SANYAL, K. 2023. Functional plasticity in chromosome-microtubule coupling on the evolutionary time scale. *Life Science Alliance,* 6, e202201720.

FINLEY, K. R. & BERMAN, J. 2005. Microtubules in *Candida albicans* hyphae drive nuclear dynamics and connect cell cycle progression to morphogenesis. *Eukaryotic Cell,* 4, 1697-1711.

GEST, H. 2004. The discovery of microorganisms by Robert Hooke and Antoni Van Leeuwenhoek, fellows of the Royal Society. *Notes Rec R Soc Lond,* 58, 187-201.

HINTERNDORFER, K., LAPORTE, M. H., MIKUS, F., TAFUR, L., BOURGOINT, C., PROUTEAU, M., DEY, G., LOEWITH, R., GUICHARD, P. & HAMEL, V. 2022. Ultrastructure expansion microscopy reveals the cellular architecture of budding and fission yeast. *J Cell Sci,* 135.

SHAH, H., OLIVETTA, M., BHICKTA, C., RONCHI, P., TRUPINIĆ, M., TROMER, E. C., TOLIĆ, I. M., SCHWAB, Y., DUDIN, O. & DEY, G. 2024. Life-cycle-coupled evolution of mitosis in close relatives of animals. *Nature*.

Second decision letter

MS ID#: JOCES/2024/262046

MS TITLE: Expansion microscopy reveals unique ultrastructural features of pathogenic budding yeast species

AUTHORS: Md Hashim Reza, Srijana Dutta, Rohit Goyal, Hiral Shah, Gautam Dey, and Kaustuv Sanyal
ARTICLE TYPE: Tools and Resources

We have now reached a decision on the above manuscript.

To see the reviewers' reports and a copy of this decision letter, please go to: https://submit-jcs.biologists.org and click on the 'Manuscripts with Decisions' queue in the Author Area. (The corresponding author only has access to reviews.)

© 2024. Published by The Company of Biologists under the terms of the Creative Commons Attribution License (https://creativecommons.org/licenses/by/4.0/).

As you will see, reviewer 1 gave a favourable report but Reviewer 2 raised some points requiring amendments to your manuscript. Please consider how to best address these comments by modifying your figures and text (no additional experiments should be required at this stage). I hope that you will be able to carry these out because I would like to be able to accept your paper.

Please ensure that you clearly highlight all changes made in the revised manuscript. Please avoid using 'Tracked changes' in Word files as these are lost in PDF conversion.

I should be grateful if you would also provide a point-by-point response detailing how you have dealt with the points raised by the reviewers in the 'Response to Reviewers' box. Please attend to all of the reviewers' comments. If you do not agree with any of their criticisms or suggestions please explain clearly why this is so.

Reviewer 1

Advance summary and potential significance to field

The work produced by Reza and colleagues delivered an great application of expansion microscopy to several fungi species, some of them medically relevant. The beautiful imaging is also appreciated. In summary, this study shows the characterisation of organelle organisation in C. albicans and other medically relevant species using expansion microscopy. To achieve this, the authors made modifications to the highly used expansion microscopy approach, U-ExM, and applied it to a diverse range of fungal organisms.

Comments for the author

No further suggestions. The rebuttal and revisions done by the authors satisfied all my previous criticisms.
Good job!

I found a typo on page 8, LINE 244 "visaulising".

Reviewer 2

Advance summary and potential significance to field

Reza et al made modifications, including new experiments, in the revised paper to address reviewers' comments. However, I still think that the revision does not go far enough to merit publication in the JCS.

Comments for the author

1) My initial concerns of over-statements such as “unique ultrastructural features” still remains.

2) The authors performed live-cell movies, and presented the data in new Fig 5.
Plot B displays spindle length versus time in mean and SEM. Length should not be plotted as SEM, as it is not standard error of means; instead length should be plotted as SD standard deviation. Also, the time x-axis has no numerical indications.

Ultimately, Fig 5 shows live-cell imaging of dynamic spindles. These data set could also be used to confirm the long astral MTs reported in the U-ExM images.

3) Finally, there is no structural information provided in Fig 6 other than other pathogenic yeasts can also be expanded using the U-ExM method.

In summary, the U-ExM method has been published before in the JCS to described subcellular features of budding and fission yeast. Here, U-ExM is applied to C. albicans, but with little new biological information to be learned.

© 2024. Published by The Company of Biologists under the terms of the Creative Commons Attribution License (https://creativecommons.org/licenses/by/4.0/).

Second revision

Author response to reviewers' comments

First revision

Author response to reviewers' comments

Point-by-point responses to reviewers' queries

Reza et al. (2024) MS# JOCES/2024/262046

Reviewer 1
Advance Summary and Potential Significance to Field:
The work produced by Reza and colleagues delivered a great application of expansion microscopy to several fungi species, some of them medically relevant. The beautiful imaging is also appreciated. In summary, this study shows the characterisation of organelle organisation in *C. albicans* and other medically relevant species using expansion microscopy. To achieve this, the authors made modifications to the highly used expansion microscopy approach, U ExM, and applied it to a diverse range of fungal organisms.

Reviewer 1 Comments for the Author:
No further suggestions. The rebuttal and revisions done by the authors satisfied all my previous criticisms. Good job!
Au: We thank Reviewer 1 for appreciating our work, image quality and acknowledging our efforts to answer all the questions during revision.

R1.1: I found a typo on page 8, LINE 244 "visaulising".
Au: We thank the reviewer for pointing this out. We have corrected this (Page 8, line 244).

Reviewer 2
Advance Summary and Potential Significance to Field:
Reza et al made modifications, including new experiments, in the revised paper to address reviewers' comments. However, I still think that the revision does not go far enough to merit publication in the JCS.

Reviewer 2 Comments for the Author:
R2.1: My initial concerns of over-statements such as "unique ultrastructural features" still remains.
Au: We have now modified the title to "Expansion microscopy reveals characteristic ultrastructural features of pathogenic budding yeast species."

R2.2: The authors performed live-cell movies, and presented the data in new Fig 5. Plot B displays spindle length versus time in mean and SEM. Length should not be plotted as SEM, as it is not standard error of means; instead, length should be plotted as SD standard deviation. Also, the time x-axis has no numerical indications. Ultimately, Fig 5 shows live-cell imaging of dynamic spindles. These data set could also be used to confirm the long astral MTs reported in the U-ExM images.
Au: We thank the reviewer for pointing this out. We concur with the reviewer and have now plotted all the individual live-cell movie data points in Figure 5B (Page 10, lines 294-296). We have also included the numerical values on the *x*-axis.

© 2024. Published by The Company of Biologists under the terms of the Creative Commons Attribution License (https://creativecommons.org/licenses/by/4.0/).

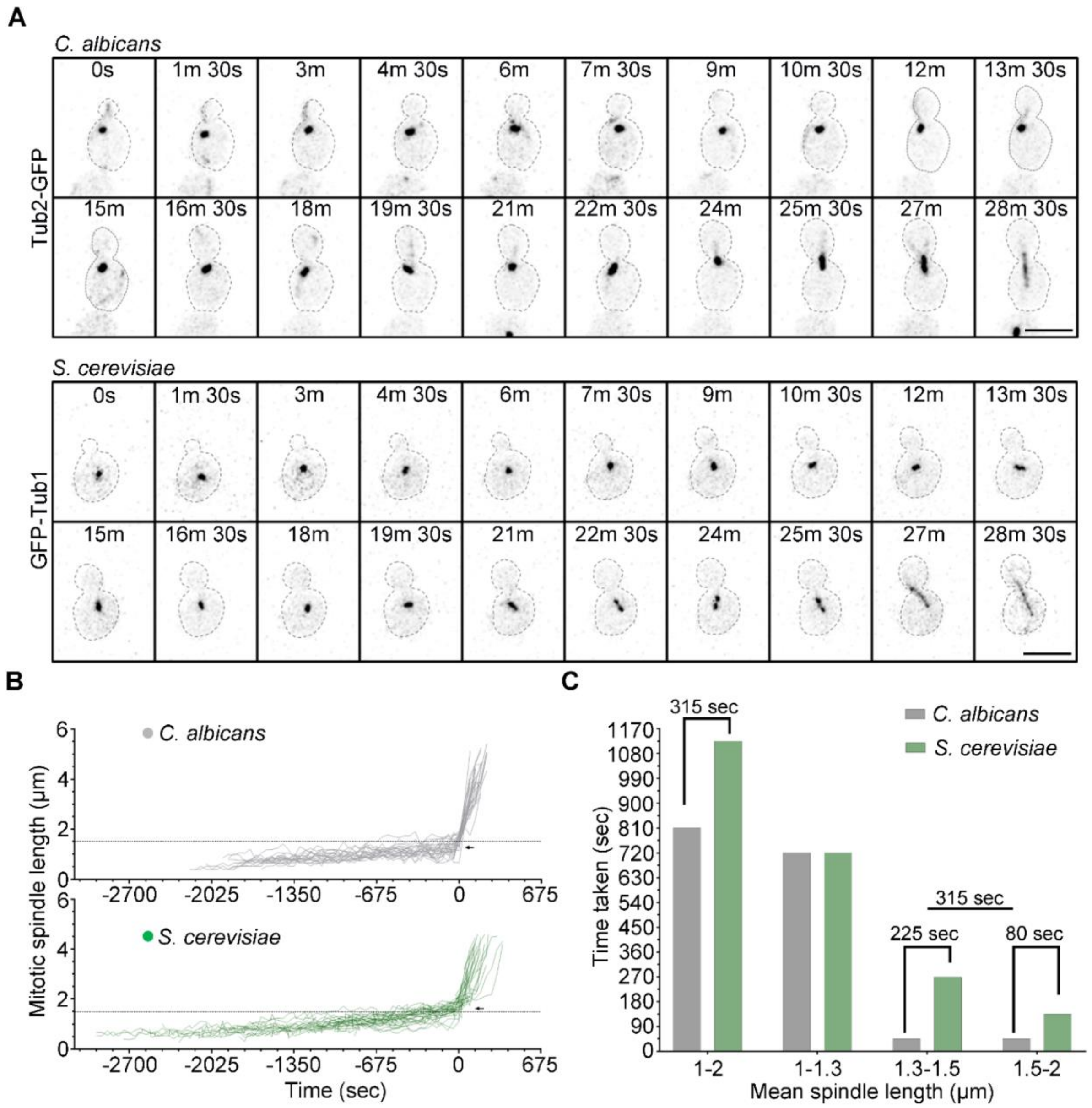

Figure R1. *C. albicans* displays a faster anaphase onset with a shorter pre-anaphase spindle. (A) A montage of time-lapse images showing dynamics of the mitotic spindle, tagged with GFP, during the cell cycle in *C. albicans* (*top*) and *S. cerevisiae* (*bottom*) at 30°C. Scale bar 5 µm. (B) Spindle length progression during cell cycle in *C. albicans* (grey) and *S. cerevisiae* (green). *n*=25 and 23 live-cell movies for *C. albicans* and *S. cerevisiae*, respectively. The horizontal line (black dotted) is drawn at 1.5 µm spindle length as a reference point. The black arrow indicates anaphase onset. The first time point corresponding to >=2 µm spindle length is set to zero to align all the live-cell movie data. (C) Bar graph displaying the time taken (*y*-axis) for reaching maximum mean spindle length in *C. albicans* (grey) and *S. cerevisiae* (green) for different categories. The difference in time taken for each category of mean spindle length between *C. albicans* and *S. cerevisiae* is mentioned in the graph.

We have now moved the plot with SEM to the supporting Figure 5 (Figure S5G) in the revised manuscript. As suggested, we have also plotted a graph with their SD and analyzed the difference in spindle length before anaphase onset for *C. albicans* and *S. cerevisiae*. Our results suggest that *C. albicans* displays a significantly shorter spindle which agrees with our previous observation. This is included as part of supporting Figure 5 (Figure S5H) in the revised manuscript (Page 10, lines 303-304).

© 2024. Published by The Company of Biologists under the terms of the Creative Commons Attribution License (https://creativecommons.org/licenses/by/4.0/).

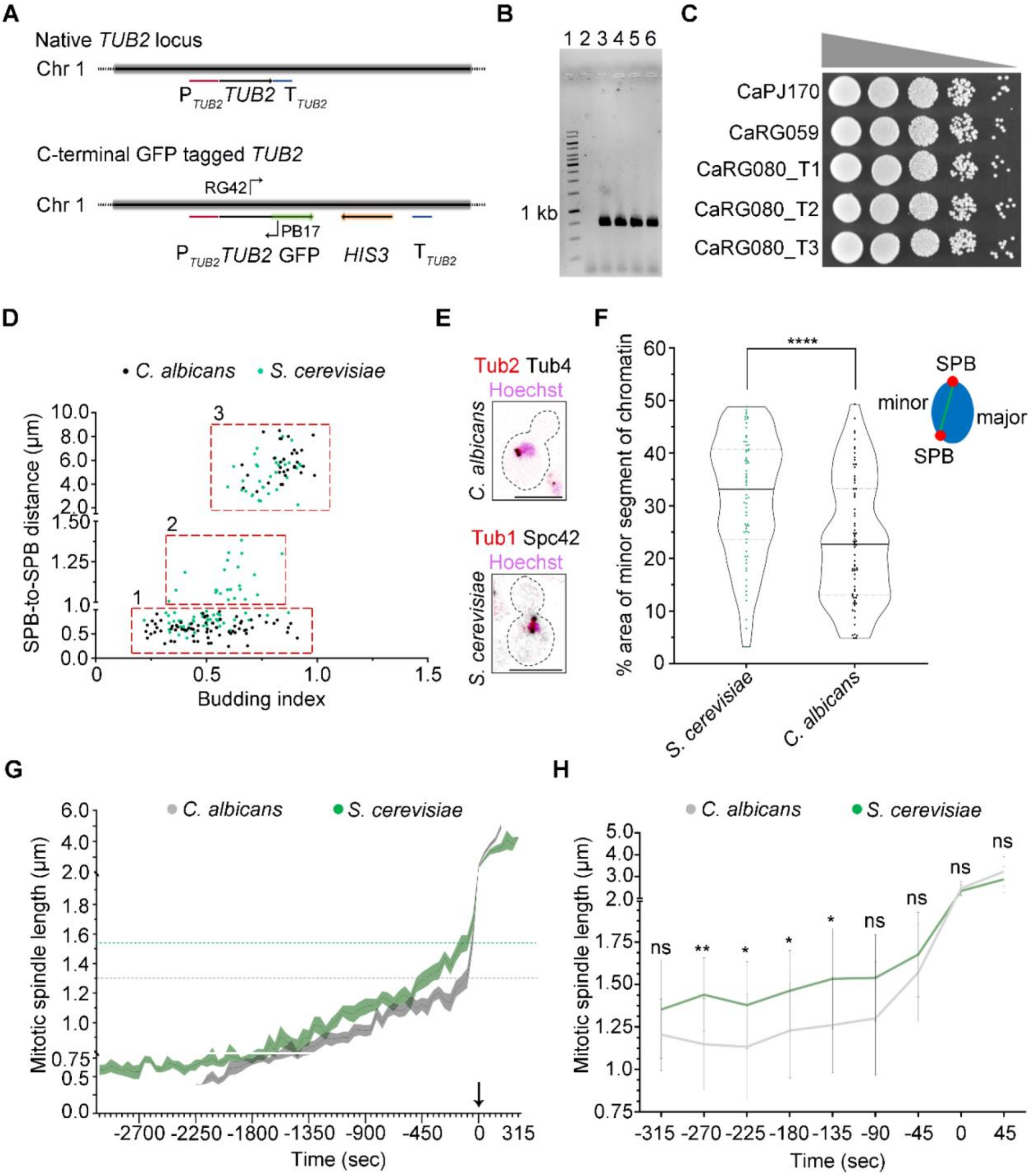

Figure R2. SPB positioning during cell division in *C. albicans*. (A) A schematic representation showing *TUB2* tagging with GFP at the C-terminus at the native locus. Primer pairs RG42/PB17 (~690 bp) were used to confirm the integration of the tagging cassette at the native locus. (B) A 1% agarose gel displaying the screening of the transformants by PCR using primer pairs RG42/PB17. Lane 1: 1 kb DNA ladder, lane 2: parent untagged strain (CaPJ170), lane 3: parent tagged with Tub2-GFP (CaRG059), lane 4-6: parent tagged with Tub2-GFP Tub4-mCherry (CaRG080_T1-T3). (C) Overnight grown *C. albicans* cells (CaPJ170, CaRG059, and CaRG080_T1-T3) were 10-fold serially diluted, and spotted on YPD+uridine. Plates were photographed after incubation at 30°C for 30 h. (D) Scatter plot displaying SPB- to-SPB distance with respect to the budding index in *C. albicans* (black) and *S. cerevisiae* (green). SPB-to-SPB distance above 2 µm represents an anaphase spindle. The red boxes represent three prominent clusters obtained in *S. cerevisiae*. *n*> 100 cells. (E) Maximum intensity projection of *S. cerevisiae*, tagged with GFP-Tub1 and Spc42-mCherry, and *C. albicans* tagged with Tub2-GFP and Tub4-mCherry and co-stained with Hoechst during the pre-anaphase stage. Scale bar 5 µm. (F) Cartoon showing the mitotic spindle (green) between the two SPBs (red)

© 2024. Published by The Company of Biologists under the terms of the Creative Commons Attribution License (https://creativecommons.org/licenses/by/4.0/).

dividing the Hoechst-stained chromatin (blue) area into two unequal segments (minor and major) as visually observed in a 2D-projected image. Violin plot showing percent area covered by the minor segment of Hoechst-stained chromatin in *C. albicans* and *S. cerevisiae*. *n*> 70 cells. Statistical analysis was done by Unpaired *t*-test with Welch's correction (****p<0.0001). (G) A line graph displaying the mitotic spindle length distribution during cell cycle progression in *C. albicans* (grey) and *S. cerevisiae* (green). Anaphase onset is marked by horizontal lines, grey (*C. albicans*) and green (*S. cerevisiae*). *n*=25 and 23 live-cell movies for *C. albicans* and *S. cerevisiae*, respectively. The first time point corresponding to >=2 µm spindle length (black arrow) was used as a reference point and is set to zero to align all the live- cell movie data. The plot represents mean (black line) ±s.e.m. (H) Representation of spindle length before and after 2 µm spindle in *C. albicans* (grey) and *S. cerevisiae* (green). Zero time point corresponds to >=2 µm spindle. The lines represent mean from 25 and 23 live-cell movies for *C. albicans* and *S. cerevisiae*, respectively. Statistical analysis was done using Two-way ANOVA followed by Sidak's multiple comparisons test (**p=0.0014, *p=0.0383/0/0269/0.0186). Error bars show mean± s.d.

We have now included the length of aMTs from live-cell movies in *C. albicans* during the pre-anaphase stage of cell division which is presented as Figure 4F in the revised manuscript. The results have been included (Page 8, lines 252-253). The analyses show that *C. albicans* aMT length at pre-anaphase ranges from 0.35 to 6.82 µm (U-ExM) and 0.36 to 5.62 µm (live-cell). The variation in mean spindle length (2.61 µm in U-ExM and 2.10 µm in live-cell) observed is likely due to a) difference in sample size, b) visualisation of dynamic shorter aMTs from live- cell movies, c) lower intensity (1%) exposure used for live-cell imaging compared to 11% exposure for U-ExM. Indeed, the analyses from both live-cell and U-ExM suggest longer aMTs in *C. albicans* in comparison to *S. cerevisiae*.

© 2024. Published by The Company of Biologists under the terms of the Creative Commons Attribution License (https://creativecommons.org/licenses/by/4.0/).

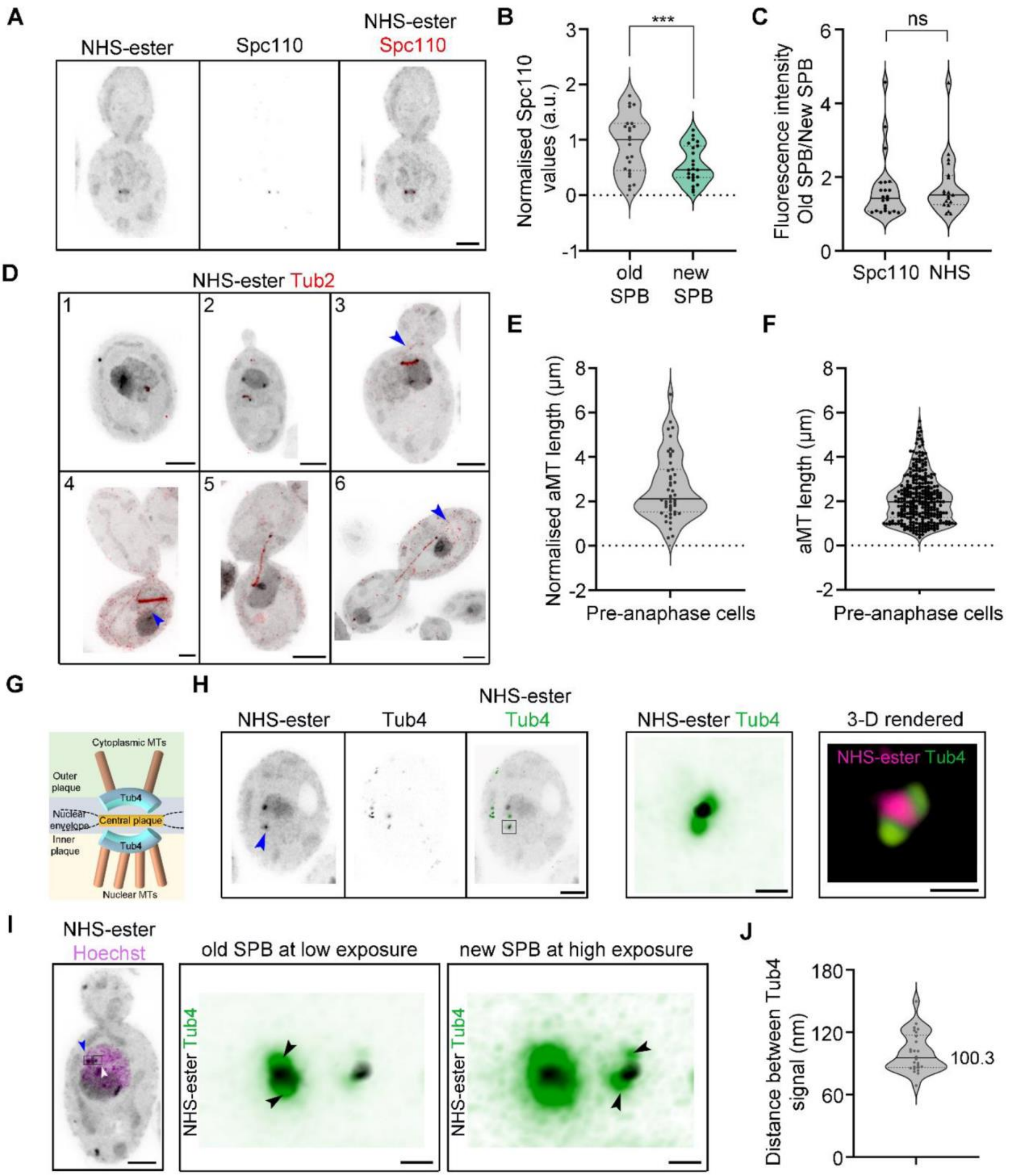

Figure R3. SPB organisation in *C. albicans*. (A) Maximum intensity projection of *C. albicans* cells, co-stained with NHS-ester (grey) and anti-GFP (Spc110-GFP, red). Scale bar 5 μm. (B) The violin plot displays Spc110 signal intensities. The signal was quantified and normalised to the mean of SPB1 (old SPB). *n*=23 expanded cells analysed. Statistical analysis was done using a paired *t*-test (***p=0.0005). (C) The violin plot displays the ratio of fluorescent intensities between old and new SPB as determined by NHS-ester signal intensities. The signal was quantified and the old SPB signal was normalised to the signal from the new SPB. *n*=22 (Spc110) and 19 (NHS-ester) expanded cells analysed. Statistical analysis was done using an Unpaired *t*-test. (D) Maximum intensity projection of *C. albicans* cells co-stained with NHS- ester (grey) and anti-GFP (Tub2-GFP, red), at various stages of the cell cycle (1, 2, 3, 4, 5, and 6). The blue arrowheads mark the astral microtubules (aMTs). Scale bar 5 μm. (E) A violin plot showing the distribution of aMT length at the pre-anaphase stage of the cell cycle in expanded *C. albicans* cells. *n*=47 aMTs analysed. (F) A violin plot showing the distribution of aMT length at the pre-anaphase stage of the cell cycle from live-cell of *C. albicans* tagged with Tub2-GFP. *n*=303 aMTs analysed from 11 live-cell movies. (G) Schematic

© 2024. Published by The Company of Biologists under the terms of the Creative Commons Attribution License (https://creativecommons.org/licenses/by/4.0/).

showing the spatial position of Tub4 at the SPB as reported from *S. cerevisiae*. (H) A representative maximum- intensity projection of *C. albicans* cells co-stained with NHS-ester (grey) and anti-GFP (Tub4- GFP, green) in unbudded cells. Scale bar 5 µm. Inset, a magnified region depicted by a black- bordered square and a 3-D rendered image of the magnified region is also shown. Scale bar 125 nm. (I) Maximum-intensity projection of *C. albicans* cells co-stained with NHS-ester (grey) and Hoechst (magenta) at the pre-anaphase stage of the cell cycle. Scale bar 5 µm. The blue and white arrowhead represents the old and new SPBs, based on signal intensity. Inset, a magnified region is shown for the two SPBs. The first inset highlights the Tub4 arrangement (black arrowheads) at the old SPB, visible at low-intensity exposure. The second inset highlights the Tub4 arrangement (black arrowheads) at the new SPB visible only at high- intensity exposure. Scale bar 125 nm. (J) A violin plot displaying the distance between two Tub4 fluorescent signals rescaled after expansion (N=2, *n*=26).

R2.3: Finally, there is no structural information provided in Fig 6 other than other pathogenic yeasts can also be expanded using the U-ExM method.

Au: We have now included the structural information for *Candida dubliniensis* and *Nakaseomyces glabratus* showing various organelles (mitochondria, SPBs, vacuoles, nucleolus, and mitotic bridge) based on pan-labelling with NHS-ester at different stages of cell cycle. This is included as Supporting Figure 6. The results have been included in the revised manuscript (Page 10, lines 323-324).

© 2024. Published by The Company of Biologists under the terms of the Creative Commons Attribution License (https://creativecommons.org/licenses/by/4.0/).

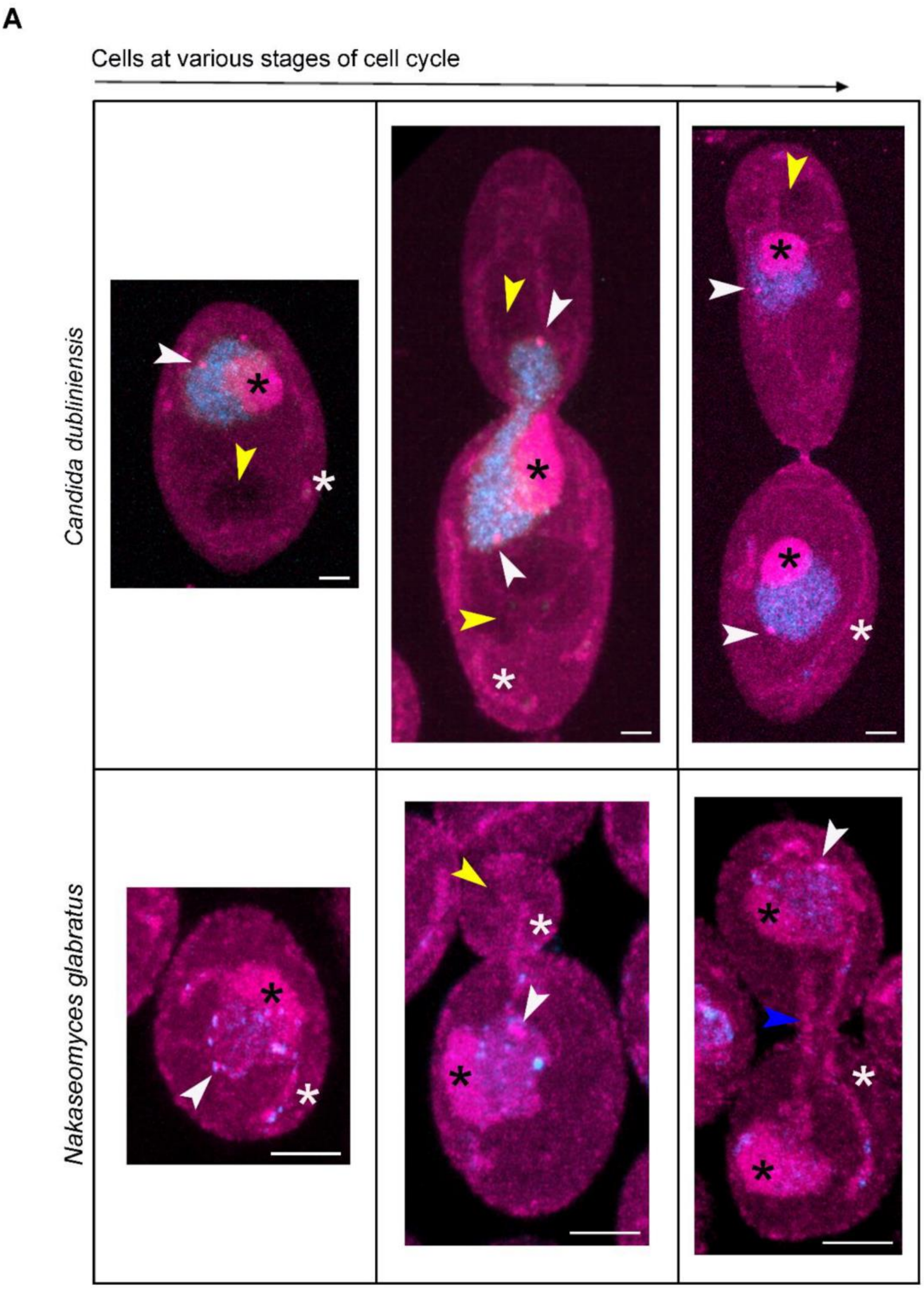

Figure R4. Pan-labelling of proteome displays sub-cellular organisation in expanded pathogenic yeast cells. (A) Representative images of *C. dubliniensis* and *N. glabratus* co- stained with NHS-ester (magenta) and Hoechst (blue) showing sub-cellular organisation at various stages of the cell cycle. The yellow, white, and blue arrowheads represent vacuole, SPBs, and mitotic bridge respectively. The white and black asterisks mark mitochondria and nucleolus, respectively. Scale bar 5 μm.

We would also like to bring this to the reviewer's notice that in our previous draft, we had shown various structural components [SPBs (white arrowheads), nucleolus and mitochondria (yellow and white asterisks, respectively)] in these expanded cells in Figure 6. Additionally, using U-ExM we also mentioned the distinct side-by-side arrangement of SPBs in two fungal pathogens, *C. dubliniensis* and *N. glabratus* (Fig. 6H-I).

© 2024. Published by The Company of Biologists under the terms of the Creative Commons Attribution License (https://creativecommons.org/licenses/by/4.0/).

R2.4: In summary, the U-ExM method has been published before in the JCS to described subcellular features of budding and fission yeast. Here, U-ExM is applied to *C. albicans*, but with little new biological information to be learned.
Au: Our results demonstrate that the successful expansion of pathogenic yeasts by U-ExM protocol required specific and significant modifications from the initial protocol for *S. cerevisiae* and *S. pombe* (Hinterndorfer et al., 2022). The utilisation of available molecular tools for understanding these human pathogens is greatly challenged by the varied range of cellular architectural differences. U-ExM enabled us to observe the distinct SPB arrangement during the pre-anaphase stage of cell division in expanded *C. albicans* cells which we could validate by live-cell analyses. We also learnt that this side-by-side SPB arrangement could be a conserved feature shared across fungal species that diverged millions of years ago.

References

HINTERNDORFER, K., LAPORTE, M. H., MIKUS, F., TAFUR, L., BOURGOINT, C., PROUTEAU, M., DEY, G., LOEWITH, R., GUICHARD, P. & HAMEL, V. 2022. Ultrastructure expansion microscopy reveals the cellular architecture of budding and fission yeast. *J Cell Sci,* 135.

Third decision letter

MS ID#: JOCES/2024/262046

MS TITLE: Expansion microscopy reveals characteristic ultrastructural features of pathogenic budding yeast species

AUTHORS: Md Hashim Reza, Srijana Dutta, Rohit Goyal, Hiral Shah, Gautam Dey, and Kaustuv Sanyal
ARTICLE TYPE: Tools and Resources

I am happy to tell you that your manuscript has been accepted for publication in Journal of Cell Science, pending standard publication integrity checks.

© 2024. Published by The Company of Biologists under the terms of the Creative Commons Attribution License (https://creativecommons.org/licenses/by/4.0/).
